# Supplementary material for: HIDE: hierarchical cell-type deconvolution
Source: Bioinformatics. 2025 Jul 15;41(Suppl 1):i207–16. doi: 10.1093/bioinformatics/btaf179 (PMC12261400; doi:10.1093/bioinformatics/btaf179)
Supplement: btaf179_Supplementary_Data [file btaf179_supplementary_data.pdf]

Supplementary Material: HIDE

1 Informations corresponding to the single cell RNA-Seq dataset

	single cells in training set	single cells in test set	single cells in BayesPrism	percentage
Arterial EC	340	341	170	0.3
Breast basal cell	1741	1741	870	1.8
Breast cancer specific luminal cell	26321	26321	13160	26.7
Breast cancer specific proliferation luminal cell	3307	3308	1653	3.4
Capillary EC	1014	1014	507	1
CD4 T	7716	7717	3858	7.8
cDC2	1166	1166	583	1.2
CFD fibroblast	1582	1583	791	1.6
CXCL1/2/3 fibroblast	2847	2848	1423	2.9
CXCL13 exhausted CD8 T	1146	1146	573	1.2
GZMH CD8 T	3893	3894	1946	4
GZMK CD8 T	4952	4952	2476	5
IgA plasma	569	569	284	0.6
IgG plasma cell	2241	2241	1120	2.3
INF responsed T	97	97	48	0.1
Luminal progenitor	2646	2646	1323	2.7
Lymphatic EC	162	162	81	0.2
Macrophage	7427	7428	3713	7.5
Mast cell	444	444	222	0.5
Monocyte	1120	1120	560	1.1
mregDC	134	134	67	0.1
NK cell	1594	1595	797	1.6
Other B cells	2514	2515	1257	2.6
Other fibroblasts	3576	3577	1788	3.6
pDC	325	325	162	0.3
Pericyte	472	472	236	0.5
Proliferation macrophage	507	507	253	0.5
Proliferation T/NK	1053	1053	526	1.1
Smooth muscle cell	1487	1487	743	1.5
Tfh	2070	2070	1035	2.1
TGM2 luminal cell	723	723	361	0.7
TNBC-specific epithelial cell	8496	8496	4248	8.6
Treg	3495	3496	1747	3.5
Venous EC	1276	1277	638	1.3
sum	98453	98465	49219	100

Table S1: **Distribution of the single cells used for benchmarking.** Distribution of single cells used to create the artificial training and test set and the number of single cells used for training with BayesPrism. The percentage remains the same for training, test and BayesPrism set.

	BayesPrism	CIBERSORTx	MuSiC	HIDE
Arterial EC	0.445 ± 0.042	0.321 ± 0.041	0.345 ± 0.041	0.643 ± 0.033
Breast basal cell	0.561 ± 0.026	0.663 ± 0.022	0.433 ± 0.012	**0.878 ± 0.005**
Breast cancer specific luminal cell	0.531 ± 0.025	0.65 ± 0.025	0.656 ± 0.025	**0.907 ± 0.006**
Breast cancer specific proliferation luminal cell	0.311 ± 0.026	0.307 ± 0.038	0.348 ± 0.031	0.625 ± 0.02
Capillary EC	0.487 ± 0.03	0.459 ± 0.018	0.275 ± 0.037	0.719 ± 0.022
CD4 T	0.612 ± 0.025	0.475 ± 0.037	0.465 ± 0.023	0.769 ± 0.011
cDC2	0.543 ± 0.017	0.4 ± 0.024	0.48 ± 0.022	0.689 ± 0.023
CFD fibroblast	0.565 ± 0.031	0.666 ± 0.016	0.501 ± 0.031	**0.812 ± 0.01**
CXCL1/2/3 fibroblast	0.696 ± 0.02	0.788 ± 0.012	0.536 ± 0.03	**0.901 ± 0.008**
CXCL13 exhausted CD8 T	0.396 ± 0.026	0.255 ± 0.026	0.296 ± 0.031	0.556 ± 0.015
GZMH CD8 T	0.424 ± 0.031	0.277 ± 0.028	0.177 ± 0.026	0.612 ± 0.022
GZMK CD8 T	0.626 ± 0.024	0.555 ± 0.029	0.366 ± 0.046	0.78 ± 0.015
IgA plasma	0.791 ± 0.01	0.75 ± 0.018	0.687 ± 0.018	**0.877 ± 0.007**
IgG plasma cell	0.757 ± 0.019	0.787 ± 0.011	0.775 ± 0.014	**0.936 ± 0.004**
INF responsed T	0.234 ± 0.048	0.206 ± 0.029	0.142 ± 0.03	0.176 ± 0.049
Luminal progenitor	0.565 ± 0.017	0.5 ± 0.021	0.484 ± 0.024	**0.838 ± 0.005**
Lymphatic EC	0.636 ± 0.033	0.488 ± 0.029	0.227 ± 0.03	0.763 ± 0.017
Macrophage	0.509 ± 0.027	0.586 ± 0.02	0.505 ± 0.024	**0.898 ± 0.005**
Mast cell	0.599 ± 0.028	0.584 ± 0.028	0.523 ± 0.036	0.745 ± 0.019
Monocyte	0.266 ± 0.03	0.209 ± 0.04	0.263 ± 0.037	0.383 ± 0.018
mregDC	0.395 ± 0.045	0.343 ± 0.035	0.368 ± 0.044	0.651 ± 0.028
NK cell	0.524 ± 0.02	0.4 ± 0.024	0.416 ± 0.021	0.757 ± 0.01
Other B cells	0.514 ± 0.03	0.492 ± 0.034	0.429 ± 0.033	**0.84 ± 0.009**
Other fibroblasts	0.706 ± 0.019	**0.843 ± 0.014**	0.64 ± 0.021	**0.909 ± 0.007**
pDC	0.624 ± 0.035	0.571 ± 0.025	0.447 ± 0.027	**0.831 ± 0.017**
Pericyte	0.327 ± 0.037	0.4 ± 0.028	0.338 ± 0.04	0.626 ± 0.031
Proliferation macrophage	0.219 ± 0.028	0.16 ± 0.036	0.206 ± 0.043	0.38 ± 0.035
Proliferation T/NK	0.271 ± 0.027	0.267 ± 0.034	0.189 ± 0.052	0.574 ± 0.027
Smooth muscle cell	0.588 ± 0.017	0.69 ± 0.017	0.234 ± 0.033	**0.865 ± 0.01**
Tfh	0.554 ± 0.016	0.574 ± 0.021	0.515 ± 0.015	0.736 ± 0.01
TGM2 luminal cell	0.342 ± 0.034	0.369 ± 0.044	0.149 ± 0.045	0.735 ± 0.017
TNBC-specific epithelial cell	0.769 ± 0.011	0.897 ± 0.006	0.517 ± 0.025	**0.945 ± 0.005**
Treg	0.622 ± 0.025	0.566 ± 0.033	0.453 ± 0.014	**0.818 ± 0.011**
Venous EC	0.597 ± 0.024	0.568 ± 0.03	0.544 ± 0.023	**0.839 ± 0.008**
mean	0.518 ± 0.003	0.502 ± 0.006	0.41 ± 0.004	0.736 ± 0.002

Table S2: **Observed Pearson's correlations on sub-minor level,** by comparing the estimated cell proportions with the true cell proportions predicted in the benchmarking study for artificial test mixtures. The error bars correspond to ±1 SD obtained over 10 simulation runs. Correlations above 0.8 are highlighted in bold.

	BayesPrism	CIBERSORTx	MuSiC	HIDE
B cell	0.653 ± 0.028	0.646 ± 0.027	0.629 ± 0.028	**0.931 ± 0.005**
CD4 T cell	0.696 ± 0.019	0.622 ± 0.027	0.604 ± 0.025	**0.884 ± 0.006**
CD8 T cell	0.614 ± 0.018	0.494 ± 0.02	0.453 ± 0.026	**0.838 ± 0.006**
Dendritic cell	0.534 ± 0.021	0.445 ± 0.031	0.476 ± 0.025	0.754 ± 0.02
Endothelial cell	0.724 ± 0.009	0.734 ± 0.007	0.654 ± 0.018	**0.946 ± 0.003**
Epithelial cell	0.685 ± 0.019	**0.865 ± 0.01**	0.711 ± 0.015	**0.962 ± 0.003**
Fibroblast	0.701 ± 0.02	**0.866 ± 0.008**	0.664 ± 0.022	**0.953 ± 0.003**
Granulocyte	0.599 ± 0.028	0.584 ± 0.028	0.523 ± 0.036	0.745 ± 0.019
Macrophage	0.552 ± 0.015	0.628 ± 0.019	0.533 ± 0.02	**0.909 ± 0.006**
Monocyte	0.266 ± 0.03	0.209 ± 0.04	0.263 ± 0.037	0.383 ± 0.018
NK cell	0.524 ± 0.02	0.4 ± 0.024	0.416 ± 0.021	0.757 ± 0.01
Perivascular cell	0.557 ± 0.022	0.708 ± 0.023	0.423 ± 0.022	**0.905 ± 0.006**
Proliferation T/NK	0.271 ± 0.027	0.267 ± 0.034	0.189 ± 0.052	0.574 ± 0.027
mean	0.567 ± 0.005	0.575 ± 0.006	0.503 ± 0.006	**0.811 ± 0.002**

Table S3: **Observed Pearson's correlations on minor level predicted via summing approach.** Pearson's correlations are calculated between true and predited cellular composition. The error bars correspond to ±1 SD obtained over 10 simulation runs. Correlations above 0.8 are highlighted in bold.

	BayesPrism	CIBERSORTx	MuSiC	HIDE
B cell	0.653 ± 0.028	0.646 ± 0.027	0.629 ± 0.028	**0.931 ± 0.005**
Endothelial cell	0.724 ± 0.009	0.734 ± 0.007	0.654 ± 0.018	**0.946 ± 0.003**
Epithelial cell	0.685 ± 0.019	**0.865 ± 0.01**	0.711 ± 0.015	**0.962 ± 0.003**
Fibroblast	0.701 ± 0.02	**0.866 ± 0.008**	0.664 ± 0.022	**0.953 ± 0.003**
Myeloid cell	0.666 ± 0.022	0.757 ± 0.014	0.655 ± 0.021	**0.965 ± 0.002**
NK cell	0.524 ± 0.02	0.4 ± 0.024	0.416 ± 0.021	0.757 ± 0.01
Perivascular cell	0.557 ± 0.022	0.708 ± 0.023	0.423 ± 0.022	**0.905 ± 0.006**
Proliferation T/NK	0.271 ± 0.027	0.267 ± 0.034	0.189 ± 0.052	0.574 ± 0.027
T cell	0.707 ± 0.02	0.701 ± 0.023	0.706 ± 0.016	**0.924 ± 0.003**
mean	0.61 ± 0.007	0.661 ± 0.007	0.561 ± 0.006	**0.88 ± 0.003**

Table S4: **Observed Pearson's correlations on major level predicted via summing approach.** Pearson's correlations are calculated between true and predited cellular composition. The error bars correspond to ±1 SD obtained over 10 simulation runs. Correlations above 0.8 are highlighted in bold.

	BayesPrism	CIBERSORTx	MuSiC	HIDE
B cell	0.507 ± 0.029	0.6 ± 0.028	0.63 ± 0.029	**0.931 ± 0.005**
CD4 T cell	0.659 ± 0.024	0.572 ± 0.022	0.51 ± 0.037	**0.884 ± 0.006**
CD8 T cell	0.564 ± 0.017	0.553 ± 0.028	0.47 ± 0.028	**0.838 ± 0.006**
Dendritic cell	0.508 ± 0.028	0.343 ± 0.039	0.434 ± 0.024	0.754 ± 0.02
Endothelial cell	0.66 ± 0.016	0.605 ± 0.019	0.588 ± 0.02	**0.946 ± 0.003**
Epithelial cell	0.657 ± 0.019	**0.865 ± 0.007**	0.689 ± 0.015	**0.962 ± 0.003**
Fibroblast	0.663 ± 0.022	**0.819 ± 0.011**	0.598 ± 0.023	**0.953 ± 0.003**
Granulocyte	0.598 ± 0.026	0.28 ± 0.037	0.504 ± 0.035	0.745 ± 0.019
Macrophage	0.546 ± 0.015	0.612 ± 0.02	0.542 ± 0.015	**0.909 ± 0.006**
Monocyte	0.255 ± 0.025	0.187 ± 0.027	0.28 ± 0.034	0.383 ± 0.018
NK cell	0.195 ± 0.04	0.34 ± 0.024	0.392 ± 0.027	0.757 ± 0.01
Perivascular cell	0.601 ± 0.016	0.642 ± 0.022	0.424 ± 0.034	**0.905 ± 0.006**
Proliferation T/NK	0.224 ± 0.039	0.307 ± 0.041	0.16 ± 0.051	0.574 ± 0.027
mean	0.51 ± 0.006	0.517 ± 0.007	0.479 ± 0.006	**0.811 ± 0.002**

Table S5: **Observed Pearson's correlations on minor level predicted via individual level approach.** Pearson's correlations are calculated between true and predited cellular composition. The error bars correspond to ±1 SD obtained over 10 simulation runs. Correlations above 0.8 are highlighted in bold.

	BayesPrism	CIBERSORTx	MuSiC	HIDE
B cell	0.512 ± 0.029	0.598 ± 0.028	0.636 ± 0.029	**0.931 ± 0.005**
Endothelial cell	0.659 ± 0.015	0.603 ± 0.019	0.595 ± 0.018	**0.946 ± 0.003**
Epithelial cell	0.662 ± 0.019	**0.878 ± 0.007**	0.686 ± 0.015	**0.962 ± 0.003**
Fibroblast	0.665 ± 0.022	**0.822 ± 0.011**	0.599 ± 0.023	**0.953 ± 0.003**
Myeloid cell	0.66 ± 0.023	**0.863 ± 0.008**	0.657 ± 0.019	**0.965 ± 0.002**
NK cell	0.399 ± 0.021	0.273 ± 0.022	0.298 ± 0.019	0.757 ± 0.01
Perivascular cell	0.599 ± 0.016	0.603 ± 0.023	0.423 ± 0.031	**0.905 ± 0.006**
Proliferation T/NK	0.231 ± 0.042	0.299 ± 0.045	0.166 ± 0.053	0.574 ± 0.027
T cell	0.653 ± 0.021	0.668 ± 0.016	0.539 ± 0.028	**0.924 ± 0.003**
mean	0.56 ± 0.008	0.623 ± 0.006	0.511 ± 0.01	**0.88 ± 0.003**

Table S6: **Observed Pearson's correlations on major level predicted via individual level approach.** Pearson's correlations are calculated between true and predited cellular composition. The error bars correspond to ±1 SD obtained over 10 simulation runs. Correlations above 0.8 are highlighted in bold.

	BayesPrism	CIBERSORTx	MuSiC	HIDE
B cell	0.594 ± 0.028	0.575 ± 0.021	0.639 ± 0.024	**1**
CD4 T cell	**0.88 ± 0.007**	**0.805 ± 0.01**	**0.863 ± 0.01**	**1**
CD8 T cell	0.79 ± 0.019	0.663 ± 0.015	0.794 ± 0.011	**1**
Dendritic cell	**0.836 ± 0.016**	0.588 ± 0.027	**0.911 ± 0.009**	**1**
Endothelial cell	**0.87 ± 0.006**	0.63 ± 0.017	**0.928 ± 0.006**	**1**
Epithelial cell	**0.955 ± 0.002**	**0.854 ± 0.01**	**0.968 ± 0.002**	**1**
Fibroblast	**0.963 ± 0.003**	**0.877 ± 0.008**	**0.94 ± 0.003**	**1**
Granulocyte	**0.995 ± 0.001**	0.678 ± 0.021	**0.908 ± 0.013**	**1**
Macrophage	**0.978 ± 0.002**	0.87 ± 0.009	**0.982 ± 0.001**	**1**
Monocyte	**0.868 ± 0.01**	0.58 ± 0.027	**0.94 ± 0.004**	**1**
NK cell	0.355 ± 0.05	0.558 ± 0.025	0.78 ± 0.015	**1**
Perivascular cell	0.795 ± 0.018	0.765 ± 0.02	0.794 ± 0.028	**1**
Proliferation T/NK	0.426 ± 0.032	0.308 ± 0.03	0.598 ± 0.019	**1**
mean	0.793 ± 0.008	0.673 ± 0.007	**0.85 ± 0.005**	1

Table S7: **Observed Pearson's correlations on the minor cellular level resulting form comparing summing and individual level approach.** The error bars correspond to ±1 SD obtained over 10 simulation runs. Correlations above 0.8 are highlighted in bold.

	BayesPrism	CIBERSORTx	MuSiC	HIDE
B cell	0.603 ± 0.028	0.575 ± 0.02	0.647 ± 0.023	**1**
Endothelial cell	0.868 ± 0.006	0.626 ± 0.019	**0.92 ± 0.006**	**1**
Epithelial cell	**0.96 ± 0.002**	0.853 ± 0.01	**0.969 ± 0.002**	**1**
Fibroblast	**0.965 ± 0.003**	0.873 ± 0.008	**0.942 ± 0.004**	**1**
Myeloid cell	**0.973 ± 0.001**	0.803 ± 0.008	**0.959 ± 0.002**	**1**
NK cell	0.638 ± 0.028	0.343 ± 0.034	0.599 ± 0.019	**1**
Perivascular cell	0.791 ± 0.018	0.737 ± 0.022	0.795 ± 0.026	**1**
Proliferation T/NK	0.45 ± 0.031	0.261 ± 0.033	0.59 ± 0.019	**1**
T cell	0.889 ± 0.008	0.715 ± 0.015	0.769 ± 0.014	**1**
mean	0.793 ± 0.007	0.643 ± 0.009	0.799 ± 0.007	**1**

Table S8: **Observed Pearson's correlations on major level resulting form comparing summing and individual level approach.** The error bars correspond to ±1 SD obtained over 10 simulation runs. Correlations above 0.8 are highlighted in bold.

1.1 Nmae results

	BayesPrism	CIBERSORTx	MuSiC	HIDE
Arterial EC	0.129 ± 0.017	0.209 ± 0.03	0.126 ± 0.017	**0.089 ± 0.012**
Breast basal cell	0.145 ± 0.016	0.132 ± 0.015	0.331 ± 0.051	**0.07 ± 0.01**
Breast cancer specific luminal cell	0.208 ± 0.017	0.159 ± 0.014	0.295 ± 0.024	**0.053 ± 0.004**
Breast cancer specific proliferation luminal cell	0.38 ± 0.045	0.276 ± 0.034	0.646 ± 0.064	0.107 ± 0.01
Capillary EC	0.134 ± 0.015	0.184 ± 0.024	0.165 ± 0.019	0.103 ± 0.012
CD4 T	0.21 ± 0.02	0.209 ± 0.021	0.506 ± 0.054	**0.084 ± 0.008**
cDC2	0.245 ± 0.021	0.208 ± 0.017	0.242 ± 0.021	0.118 ± 0.012
CFD fibroblast	0.147 ± 0.023	0.124 ± 0.016	0.172 ± 0.026	**0.084 ± 0.011**
CXCL1/2/3 fibroblast	0.131 ± 0.017	0.1 ± 0.011	0.141 ± 0.015	**0.058 ± 0.007**
CXCL13 exhausted CD8 T	0.157 ± 0.019	0.264 ± 0.035	0.627 ± 0.075	0.131 ± 0.014
GZMH CD8 T	0.23 ± 0.023	0.343 ± 0.036	0.313 ± 0.033	0.111 ± 0.01
GZMK CD8 T	0.185 ± 0.021	0.163 ± 0.018	0.256 ± 0.025	**0.086 ± 0.01**
IgA plasma	0.123 ± 0.016	**0.078 ± 0.011**	**0.094 ± 0.014**	**0.06 ± 0.009**
IgG plasma cell	0.102 ± 0.012	**0.098 ± 0.012**	0.121 ± 0.015	**0.052 ± 0.006**
INF responsed T	0.383 ± 0.06	0.262 ± 0.041	0.836 ± 0.13	0.101 ± 0.014
Luminal progenitor	0.186 ± 0.017	0.181 ± 0.018	0.246 ± 0.027	**0.076 ± 0.008**
Lymphatic EC	**0.073 ± 0.013**	0.125 ± 0.022	0.127 ± 0.025	**0.059 ± 0.011**
Macrophage	0.221 ± 0.011	0.165 ± 0.011	0.236 ± 0.012	**0.059 ± 0.004**
Mast cell	**0.089 ± 0.01**	0.123 ± 0.017	0.106 ± 0.012	**0.094 ± 0.013**
Monocyte	0.479 ± 0.066	0.399 ± 0.045	0.541 ± 0.069	**0.143 ± 0.018**
mregDC	0.145 ± 0.031	0.176 ± 0.033	**0.07 ± 0.014**	**0.058 ± 0.012**
NK cell	0.147 ± 0.016	0.214 ± 0.026	0.271 ± 0.03	**0.097 ± 0.011**
Other B cells	0.201 ± 0.025	0.192 ± 0.028	0.279 ± 0.036	**0.082 ± 0.011**
Other fibroblasts	0.124 ± 0.009	**0.083 ± 0.007**	0.191 ± 0.014	**0.057 ± 0.004**
pDC	**0.088 ± 0.013**	0.137 ± 0.018	0.17 ± 0.023	**0.066 ± 0.01**
Pericyte	0.163 ± 0.027	0.169 ± 0.029	0.137 ± 0.025	**0.098 ± 0.018**
Proliferation macrophage	0.513 ± 0.047	0.352 ± 0.027	0.137 ± 0.015	0.139 ± 0.013
Proliferation T/NK	0.202 ± 0.022	0.302 ± 0.035	0.21 ± 0.022	0.13 ± 0.014
Smooth muscle cell	0.149 ± 0.018	0.119 ± 0.014	0.223 ± 0.027	**0.073 ± 0.009**
Tfh	0.159 ± 0.022	0.161 ± 0.02	0.248 ± 0.036	**0.099 ± 0.012**
TGM2 luminal cell	0.225 ± 0.032	0.233 ± 0.03	0.178 ± 0.023	**0.093 ± 0.012**
TNBC-specific epithelial cell	0.312 ± 0.027	**0.072 ± 0.006**	0.343 ± 0.029	**0.04 ± 0.004**
Treg	0.163 ± 0.013	0.163 ± 0.015	0.23 ± 0.016	**0.079 ± 0.006**
Venous EC	0.127 ± 0.02	0.145 ± 0.024	0.143 ± 0.02	**0.079 ± 0.012**
mean	0.196 ± 0.005	0.186 ± 0.004	0.263 ± 0.007	**0.086 ± 0.002**

Table S9: **NMAE error on sub cellular level.** Received by comparing the estimated cell proportions with the true cell proportions on the artificial test mixtures. The error bars correspond to ±1 SD obtained over 10 simulation runs. Values below 0.1 are highlighted in bold.

	BayesPrism	CIBERSORTx	MuSiC	HIDE
B cell	0.146 ± 0.013	0.131 ± 0.012	0.183 ± 0.017	**0.047 ± 0.004**
CD4 T cell	0.209 ± 0.015	0.133 ± 0.01	0.38 ± 0.024	**0.058 ± 0.004**
CD8 T cell	0.265 ± 0.019	0.179 ± 0.011	0.205 ± 0.014	**0.069 ± 0.005**
Dendritic cell	0.226 ± 0.031	0.207 ± 0.032	0.231 ± 0.029	**0.099 ± 0.015**
Endothelial cell	0.113 ± 0.011	0.132 ± 0.012	0.134 ± 0.013	**0.046 ± 0.005**
Epithelial cell	0.297 ± 0.026	0.122 ± 0.011	0.176 ± 0.017	**0.035 ± 0.003**
Fibroblast	0.132 ± 0.011	0.087 ± 0.007	0.167 ± 0.014	**0.038 ± 0.003**
Granulocyte	**0.089 ± 0.01**	0.123 ± 0.017	0.106 ± 0.012	**0.094 ± 0.013**
Macrophage	0.219 ± 0.014	0.141 ± 0.009	0.237 ± 0.014	**0.054 ± 0.004**
Monocyte	0.479 ± 0.066	0.399 ± 0.045	0.541 ± 0.069	0.143 ± 0.018
NK cell	0.147 ± 0.016	0.214 ± 0.026	0.271 ± 0.03	**0.097 ± 0.011**
Perivascular cell	0.14 ± 0.015	0.114 ± 0.009	0.2 ± 0.022	**0.061 ± 0.007**
Proliferation T/NK	0.202 ± 0.022	0.302 ± 0.035	0.21 ± 0.022	0.13 ± 0.014
mean	0.205 ± 0.008	0.176 ± 0.006	0.234 ± 0.007	**0.075 ± 0.003**

Table S10: **NMAE values on minor cellular level predicted via summing approach.** Received by comparing the estimated cell proportions with the true cell proportions on the artificial test mixtures. The error bars correspond to ±1 SD obtained over 10 simulation runs. Values below 0.1 are highlighted in bold.

	BayesPrism	CIBERSORTx	MuSiC	HIDE
B cell	0.146 ± 0.013	0.131 ± 0.012	0.183 ± 0.017	**0.047 ± 0.004**
Endothelial cell	0.113 ± 0.011	0.132 ± 0.012	0.134 ± 0.013	**0.046 ± 0.005**
Epithelial cell	0.297 ± 0.026	0.122 ± 0.011	0.176 ± 0.017	**0.035 ± 0.003**
Fibroblast	0.132 ± 0.011	**0.087 ± 0.007**	0.167 ± 0.014	**0.038 ± 0.003**
Myeloid cell	0.235 ± 0.021	0.128 ± 0.013	0.164 ± 0.015	**0.033 ± 0.003**
NK cell	0.147 ± 0.016	0.214 ± 0.026	0.271 ± 0.03	**0.097 ± 0.011**
Perivascular cell	0.14 ± 0.015	0.114 ± 0.009	0.2 ± 0.022	**0.061 ± 0.007**
Proliferation T/NK	0.202 ± 0.022	0.302 ± 0.035	0.21 ± 0.022	0.13 ± 0.014
T cell	0.355 ± 0.024	0.111 ± 0.008	0.265 ± 0.017	**0.047 ± 0.003**
mean	0.196 ± 0.006	0.149 ± 0.005	0.197 ± 0.006	**0.059 ± 0.002**

Table S11: **NMAE values on major cellular level predicted via summing approach.** Received by comparing the estimated cell proportions with the true cell proportions on the artificial test mixtures. The error bars correspond to ±1 SD obtained over 10 simulation runs. Values below 0.1 are highlighted in bold.

	BayesPrism	CIBERSORTx	MuSiC	HIDE
B cell	0.218 ± 0.021	0.168 ± 0.016	0.214 ± 0.02	**0.047 ± 0.004**
CD4 T cell	0.27 ± 0.018	0.171 ± 0.012	0.293 ± 0.022	**0.058 ± 0.004**
CD8 T cell	0.287 ± 0.02	0.175 ± 0.015	0.283 ± 0.018	**0.069 ± 0.005**
Dendritic cell	0.213 ± 0.029	0.273 ± 0.043	0.224 ± 0.03	**0.099 ± 0.015**
Endothelial cell	0.132 ± 0.015	0.155 ± 0.017	0.139 ± 0.015	**0.046 ± 0.005**
Epithelial cell	0.319 ± 0.028	**0.089 ± 0.009**	0.231 ± 0.021	**0.035 ± 0.003**
Fibroblast	0.142 ± 0.012	**0.093 ± 0.007**	0.201 ± 0.017	**0.038 ± 0.003**
Granulocyte	**0.089 ± 0.01**	0.311 ± 0.047	0.126 ± 0.013	**0.094 ± 0.013**
Macrophage	0.208 ± 0.013	0.161 ± 0.01	0.219 ± 0.014	**0.054 ± 0.004**
Monocyte	0.535 ± 0.073	0.472 ± 0.063	0.566 ± 0.07	0.143 ± 0.018
NK cell	0.21 ± 0.027	0.281 ± 0.035	0.379 ± 0.042	**0.097 ± 0.011**
Perivascular cell	0.14 ± 0.016	0.146 ± 0.014	0.213 ± 0.024	**0.061 ± 0.007**
Proliferation T/NK	0.39 ± 0.046	0.29 ± 0.033	0.368 ± 0.045	0.13 ± 0.014
mean	0.242 ± 0.009	0.214 ± 0.009	0.266 ± 0.009	**0.075 ± 0.003**

Table S12: **NMAE values on minor cellular level predicted via independent level approach.** Received by comparing the estimated cell proportions with the true cell proportions on the artificial test mixtures. The error bars correspond to ±1 SD obtained over 10 simulation runs. Values below 0.1 are highlighted in bold.

	BayesPrism	CIBERSORTx	MuSiC	HIDE
B cell	0.208 ± 0.02	0.173 ± 0.016	0.217 ± 0.021	**0.047 ± 0.004**
Endothelial cell	0.131 ± 0.015	0.159 ± 0.018	0.137 ± 0.014	**0.046 ± 0.005**
Epithelial cell	0.273 ± 0.024	**0.071 ± 0.007**	0.245 ± 0.022	**0.035 ± 0.003**
Fibroblast	0.145 ± 0.013	**0.091 ± 0.007**	0.201 ± 0.017	**0.038 ± 0.003**
Myeloid cell	0.242 ± 0.021	**0.075 ± 0.005**	0.165 ± 0.014	**0.033 ± 0.003**
NK cell	0.164 ± 0.018	0.337 ± 0.04	0.425 ± 0.046	**0.097 ± 0.011**
Perivascular cell	0.141 ± 0.016	0.165 ± 0.015	0.214 ± 0.024	**0.061 ± 0.007**
Proliferation T/NK	0.382 ± 0.043	0.305 ± 0.035	0.382 ± 0.047	0.13 ± 0.014
T cell	0.386 ± 0.024	0.136 ± 0.01	0.33 ± 0.021	**0.047 ± 0.003**
mean	0.23 ± 0.007	0.168 ± 0.007	0.257 ± 0.009	**0.059 ± 0.002**

Table S13: **NMAE values on major cellular levelpredicted via independent level approach.** Received by comparing the estimated cell proportions with the true cell proportions on the artificial test mixtures. The error bars correspond to ±1 SD obtained over 10 simulation runs. Values below 0.1 are highlighted in bold.

2 Ablation study

	Ablation model 1	Ablation model 2	HIDE
Arterial EC	0.676 ± 0.024	0.618 ± 0.025	0.643 ± 0.033
Breast basal cell	**0.884 ± 0.006**	**0.861 ± 0.008**	**0.878 ± 0.005**
Breast cancer specific luminal cell	**0.862 ± 0.008**	**0.856 ± 0.01**	**0.907 ± 0.006**
Breast cancer specific proliferation luminal cell	0.601 ± 0.026	0.602 ± 0.02	0.625 ± 0.02
Capillary EC	0.687 ± 0.03	0.655 ± 0.029	0.719 ± 0.022
CD4 T	0.77 ± 0.01	0.734 ± 0.012	0.769 ± 0.011
cDC2	0.666 ± 0.016	0.69 ± 0.013	0.689 ± 0.023
CFD fibroblast	0.796 ± 0.009	**0.805 ± 0.01**	**0.812 ± 0.01**
CXCL1/2/3 fibroblast	**0.891 ± 0.008**	**0.89 ± 0.009**	**0.901 ± 0.008**
CXCL13 exhausted CD8 T	0.54 ± 0.019	0.55 ± 0.021	0.556 ± 0.015
GZMH CD8 T	0.588 ± 0.019	0.58 ± 0.024	0.612 ± 0.022
GZMK CD8 T	0.757 ± 0.014	0.746 ± 0.017	0.78 ± 0.015
IgA plasma	**0.888 ± 0.006**	**0.888 ± 0.006**	**0.877 ± 0.007**
IgG plasma cell	**0.949 ± 0.004**	**0.937 ± 0.02**	**0.936 ± 0.004**
INF responsed T	0.308 ± 0.035	0.27 ± 0.016	0.176 ± 0.049
Luminal progenitor	**0.838 ± 0.007**	**0.828 ± 0.007**	**0.838 ± 0.005**
Lymphatic EC	**0.814 ± 0.022**	0.749 ± 0.02	0.763 ± 0.017
Macrophage	**0.809 ± 0.015**	**0.828 ± 0.01**	**0.898 ± 0.005**
Mast cell	0.759 ± 0.017	0.75 ± 0.016	0.745 ± 0.019
Monocyte	0.358 ± 0.021	0.326 ± 0.023	0.383 ± 0.018
mregDC	0.741 ± 0.025	0.668 ± 0.022	0.651 ± 0.028
NK cell	0.741 ± 0.012	0.755 ± 0.01	0.757 ± 0.01
Other B cells	**0.857 ± 0.009**	**0.839 ± 0.01**	**0.84 ± 0.009**
Other fibroblasts	**0.903 ± 0.009**	**0.902 ± 0.008**	**0.909 ± 0.007**
pDC	**0.919 ± 0.007**	**0.879 ± 0.006**	**0.831 ± 0.017**
Pericyte	0.673 ± 0.016	0.617 ± 0.017	0.626 ± 0.031
Proliferation macrophage	0.362 ± 0.026	0.379 ± 0.045	0.38 ± 0.035
Proliferation T/NK	0.557 ± 0.025	0.574 ± 0.026	0.574 ± 0.027
Smooth muscle cell	**0.855 ± 0.01**	**0.827 ± 0.01**	**0.865 ± 0.01**
Tfh	0.749 ± 0.011	0.748 ± 0.01	0.736 ± 0.01
TGM2 luminal cell	0.734 ± 0.017	0.722 ± 0.014	0.735 ± 0.017
TNBC-specific epithelial cell	**0.938 ± 0.006**	**0.934 ± 0.007**	**0.945 ± 0.005**
Treg	**0.828 ± 0.012**	**0.814 ± 0.012**	**0.818 ± 0.011**
Venous EC	**0.822 ± 0.011**	**0.823 ± 0.009**	**0.839 ± 0.008**
mean	0.739 ± 0.002	0.725 ± 0.002	0.736 ± 0.002

Table S14: **Observed Pearson's correlations on sub cellular level,** by comparing the estimated cell proportions with the true cell proportions predicted in the benchmarking study for artificial test mixtures. The error bars correspond to ±1 SD obtained over 10 simulation runs. Correlations above 0.8 are highlighted in bold.

	Ablation model 1	Ablation model 2	HIDE
B cell	**0.928 ± 0.005**	**0.92 ± 0.007**	**0.931 ± 0.005**
CD4 T cell	**0.868 ± 0.008**	**0.817 ± 0.008**	**0.884 ± 0.006**
CD8 T cell	0.783 ± 0.009	0.768 ± 0.011	**0.838 ± 0.006**
Dendritic cell	0.722 ± 0.017	0.73 ± 0.015	0.754 ± 0.02
Endothelial cell	**0.907 ± 0.005**	**0.897 ± 0.005**	**0.946 ± 0.003**
Epithelial cell	**0.947 ± 0.004**	**0.927 ± 0.005**	**0.962 ± 0.003**
Fibroblast	**0.945 ± 0.003**	**0.934 ± 0.004**	**0.953 ± 0.003**
Granulocyte	0.759 ± 0.017	0.75 ± 0.016	0.745 ± 0.019
Macrophage	**0.801 ± 0.013**	**0.868 ± 0.008**	**0.909 ± 0.006**
Monocyte	0.358 ± 0.021	0.326 ± 0.023	0.383 ± 0.018
NK cell	0.741 ± 0.011	0.755 ± 0.01	0.757 ± 0.01
Perivascular cell	**0.891 ± 0.006**	**0.846 ± 0.013**	**0.905 ± 0.006**
Proliferation T/NK	0.555 ± 0.025	0.574 ± 0.026	0.574 ± 0.027
mean	0.785 ± 0.004	0.778 ± 0.002	**0.811 ± 0.002**

Table S15: **Observed Pearson's correlations for the minor cellular level predicted via summing approach.** Pearson's correlations are calculated between true and predited cellular composition. The error bars correspond to ±1 SD obtained over 10 simulation runs. Correlations above 0.8 are highlighted in bold.

	Ablation model 1	Ablation model 2	HIDE
B cell	**0.928 ± 0.005**	**0.92 ± 0.007**	**0.931 ± 0.005**
Endothelial cell	**0.907 ± 0.005**	**0.897 ± 0.005**	**0.946 ± 0.003**
Epithelial cell	**0.947 ± 0.004**	**0.927 ± 0.005**	**0.962 ± 0.003**
Fibroblast	**0.945 ± 0.003**	**0.934 ± 0.004**	**0.953 ± 0.003**
Myeloid cell	**0.887 ± 0.007**	**0.754 ± 0.013**	**0.965 ± 0.002**
NK cell	0.741 ± 0.011	0.755 ± 0.01	0.757 ± 0.01
Perivascular cell	**0.891 ± 0.006**	**0.846 ± 0.013**	**0.905 ± 0.006**
Proliferation T/NK	0.555 ± 0.025	0.574 ± 0.026	0.574 ± 0.027
T cell	**0.892 ± 0.007**	**0.886 ± 0.006**	**0.924 ± 0.003**
mean	**0.855 ± 0.004**	**0.833 ± 0.004**	**0.88 ± 0.003**

Table S16: **Observed Pearson's correlations for the major cellular level predicted via summing approach.** Pearson's correlations are calculated between true and predited cellular composition. The error bars correspond to ±1 SD obtained over 10 simulation runs. Correlations above 0.8 are highlighted in bold.

	Ablation model 1	Ablation model 2	HIDE
Arterial EC	**0.093 ± 0.012**	0.25 ± 0.036	**0.089 ± 0.012**
Breast basal cell	**0.07 ± 0.01**	**0.099 ± 0.015**	**0.07 ± 0.01**
Breast cancer specific luminal cell	**0.076 ± 0.006**	0.464 ± 0.036	**0.053 ± 0.004**
Breast cancer specific proliferation luminal cell	0.153 ± 0.016	0.113 ± 0.012	0.107 ± 0.01
Capillary EC	0.113 ± 0.014	0.333 ± 0.039	0.103 ± 0.012
CD4 T	0.106 ± 0.01	0.117 ± 0.012	**0.084 ± 0.008**
cDC2	0.138 ± 0.012	0.996 ± 0.097	0.118 ± 0.012
CFD fibroblast	**0.095 ± 0.013**	0.123 ± 0.016	**0.084 ± 0.011**
CXCL1/2/3 fibroblast	**0.064 ± 0.008**	**0.089 ± 0.01**	**0.058 ± 0.007**
CXCL13 exhausted CD8 T	0.163 ± 0.022	0.129 ± 0.014	0.131 ± 0.014
GZMH CD8 T	0.161 ± 0.015	0.167 ± 0.014	0.111 ± 0.01
GZMK CD8 T	0.106 ± 0.012	0.153 ± 0.017	**0.086 ± 0.01**
IgA plasma	**0.052 ± 0.008**	**0.081 ± 0.012**	**0.06 ± 0.009**
IgG plasma cell	**0.042 ± 0.005**	0.152 ± 0.018	**0.052 ± 0.006**
INF responsed T	0.198 ± 0.032	0.883 ± 0.135	0.101 ± 0.014
Luminal progenitor	**0.084 ± 0.009**	0.105 ± 0.009	**0.076 ± 0.008**
Lymphatic EC	**0.067 ± 0.013**	0.381 ± 0.076	**0.059 ± 0.011**
Macrophage	**0.09 ± 0.005**	**0.098 ± 0.006**	**0.059 ± 0.004**
Mast cell	**0.09 ± 0.011**	0.169 ± 0.026	**0.094 ± 0.013**
Monocyte	0.231 ± 0.026	0.509 ± 0.057	0.143 ± 0.018
mregDC	**0.072 ± 0.016**	0.495 ± 0.098	**0.058 ± 0.012**
NK cell	0.112 ± 0.012	0.15 ± 0.016	0.**0.097 ± 0.011**
Other B cells	**0.077 ± 0.009**	0.275 ± 0.038	**0.082 ± 0.011**
Other fibroblasts	**0.062 ± 0.005**	**0.098 ± 0.006**	**0.057 ± 0.004**
pDC	**0.043 ± 0.006**	0.387 ± 0.05	**0.066 ± 0.01**
Pericyte	0.108 ± 0.017	0.424 ± 0.073	**0.098 ± 0.018**
Proliferation macrophage	0.202 ± 0.017	0.461 ± 0.041	0.139 ± 0.013
Proliferation T/NK	0.171 ± 0.019	0.139 ± 0.015	0.13 ± 0.014
Smooth muscle cell	**0.076 ± 0.01**	0.309 ± 0.036	**0.073 ± 0.009**
Tfh	0.115 ± 0.014	0.104 ± 0.012	**0.099 ± 0.012**
TGM2 luminal cell	0.101 ± 0.013	**0.097 ± 0.013**	**0.093 ± 0.012**
TNBC-specific epithelial cell	**0.045 ± 0.004**	0.187 ± 0.016	**0.04 ± 0.004**
Treg	**0.088 ± 0.006**	0.102 ± 0.007	**0.079 ± 0.006**
Venous EC	**0.083 ± 0.014**	0.118 ± 0.017	**0.079 ± 0.012**
mean	0.104 ± 0.003	0.258 ± 0.007	**0.086 ± 0.002**

Table S17: **NMAE error on sub cellular level.** Received by comparing the estimated cell proportions with the true cell proportions on the artificial test mixtures. The error bars correspond to ±1 SD obtained over 10 simulation runs. Values below 0.1 are highlighted in bold.

	Ablation model 1	Ablation model 2	HIDE
B cell	**0.05 ± 0.004**	0.244 ± 0.034	**0.047 ± 0.004**
CD4 T cell	**0.072 ± 0.005**	0.391 ± 0.034	**0.058 ± 0.004**
CD8 T cell	**0.095 ± 0.007**	0.247 ± 0.009	**0.069 ± 0.005**
Dendritic cell	0.126 ± 0.017	2.229 ± 0.212	**0.099 ± 0.015**
Endothelial cell	**0.07 ± 0.008**	0.745 ± 0.047	**0.046 ± 0.005**
Epithelial cell	**0.045 ± 0.004**	1.821 ± 0.126	**0.035 ± 0.003**
Fibroblast	**0.043 ± 0.003**	0.305 ± 0.023	**0.038 ± 0.003**
Granulocyte	**0.094 ± 0.012**	0.254 ± 0.036	**0.094 ± 0.013**
Macrophage	0.1 ± 0.008	0.269 ± 0.024	**0.054 ± 0.004**
Monocyte	0.239 ± 0.027	0.421 ± 0.034	0.143 ± 0.018
NK cell	0.116 ± 0.012	0.314 ± 0.044	**0.097 ± 0.011**
Perivascular cell	**0.072 ± 0.008**	1.088 ± 0.082	**0.061 ± 0.007**
Proliferation T/NK	0.178 ± 0.02	0.243 ± 0.029	0.13 ± 0.014
mean	0.1 ± 0.003	0.659 ± 0.026	**0.075 ± 0.003**

Table S18: **NMAE error on minor cellular level predicted via summing approach.** Received by comparing the estimated cell proportions with the true cell proportions on the artificial test mixtures. The error bars correspond to ±1 SD obtained over 10 simulation runs. Values below 0.1 are highlighted in bold.

	Ablation model 1	Ablation model 2	HIDE
B cell	**0.05 ± 0.004**	0.244 ± 0.034	**0.047 ± 0.004**
Endothelial cell	**0.07 ± 0.008**	0.745 ± 0.047	**0.046 ± 0.005**
Epithelial cell	**0.045 ± 0.004**	1.821 ± 0.126	**0.035 ± 0.003**
Fibroblast	**0.043 ± 0.003**	0.305 ± 0.023	**0.038 ± 0.003**
Myeloid cell	**0.088 ± 0.008**	1.293 ± 0.085	**0.033 ± 0.003**
NK cell	0.116 ± 0.012	0.314 ± 0.044	**0.097 ± 0.011**
Perivascular cell	**0.072 ± 0.008**	1.088 ± 0.082	**0.061 ± 0.007**
Proliferation T/NK	0.178 ± 0.02	0.243 ± 0.029	0.13 ± 0.014
T cell	**0.066 ± 0.004**	0.201 ± 0.011	**0.047 ± 0.003**
mean	**0.081 ± 0.003**	0.695 ± 0.028	**0.059 ± 0.002**

Table S19: **NMAE error on major cellular level predicted via summing approach.** Received by comparing the estimated cell proportions with the true cell proportions on the artificial test mixtures. The error bars correspond to ±1 SD obtained over 10 simulation runs. Values below 0.1 are highlighted in bold.

3 TCGA HIDE

	all subtypes	HER2	LumA	LumB	TNBC
B cell	-12.69	-15.16	5.46	-6.73	-5.85
Endothelial cell	5.98	6.25	0.96	-2.45	1.29
Epithelial cell	0.58	**7.04**	-0.01	0.49	0.68
Fibroblast	0.33	**-9.20**	0.44	**5.36**	0.38
Myeloid cell	-1.76	**-16.55**	-5.15	1.28	-3.17
NK cell	-11.65	**-41.99**	-15.93	**-37.69**	**-21.05**
Perivascular cell	4.34	3.65	**8.65**	-2.39	**6.13**
Proliferation T/NK	-15.45	3.99	-6.23	1.09	-4.01
T cell	0.21	-7.10	-1.62	-2.85	-1.69

Table S20: **Coefficients of the Cox proportional hazard model** for all major celltypes. Coefficients having a p-value smaller than 0.05 are highlighted in bold.

	all subtypes	HER2	LumA	LumB	TNBC
B cell	0.32	0.35	0.56	0.54	0.33
Endothelial cell	0.26	0.43	0.65	0.55	0.45
Epithelial cell	0.74	**0.0015**	0.99	0.68	0.33
Fibroblast	0.91	**0.047**	0.86	**0.033**	0.79
Myeloid cell	0.66	**0.0095**	0.24	0.77	0.19
NK cell	0.07	**0.037**	0.19	**0.02**	**0.0013**
Perivascular cell	0.59	0.81	**1.97e-06**	0.69	**0.013**
Proliferation T/NK	0.34	0.75	0.72	0.86	0.50
T cell	0.91	0.14	0.43	0.14	0.14

Table S21: **p-values of the Cox proportional hazard model** for all major celltypes. p-Values below 0.05 are highlighted in bold.

	all subtypes	HER2	LumA	LumB	TNBC
B cell	-12.69	-15.16	5.46	-6.73	-5.85
CD4 T cell	7.30	-3.59	**-10.10**	-0.95	-1.72
CD8 T cell	-1.53	-8.09	0.46	-3.82	-1.59
Dendritic cell	**-32.16**	-16.87	-18.52	-17.40	**-23.16**
Endothelial cell	5.98	6.25	0.96	-2.45	1.29
Epithelial cell	0.58	**7.04**	-0.01	0.49	0.68
Fibroblast	0.33	**-9.20**	0.44	**5.36**	0.38
Granulocyte	34.17	**-146.24**	-71.30	85.01	-14.90
Macrophage	0.17	**-20.31**	-5.08	4.64	-1.48
Monocyte	7.20	-240.64	-66.99	-101.17	-20.30
NK cell	-11.65	**-41.99**	-15.93	**-37.69**	**-21.05**
Perivascular cell	4.34	3.65	**8.65**	-2.39	**6.13**
Proliferation T/NK	-15.45	3.99	-6.23	1.09	-4.01

Table S22: **Coefficients of the Cox proportional hazard model** for all minor celltypes. Coefficients having a p-Value below 0.05 are highlighted in bold.

	all subtypes	HER2	LumA	LumB	TNBC
B cell	0.32	0.35	0.56	0.54	0.33
CD4 T cell	0.053	0.64	**0.023**	0.86	0.54
CD8 T cell	0.47	0.12	0.78	0.15	0.18
Dendritic cell	**0.017**	0.48	0.18	0.24	**0.0031**
Endothelial cell	0.26	0.43	0.65	0.55	0.45
Epithelial cell	0.74	**0.0015**	0.99	0.68	0.33
Fibroblast	0.91	**0.047**	0.86	**0.033**	0.79
Granulocyte	0.54	**0.024**	0.14	0.17	0.62
Macrophage	0.97	**0.033**	0.43	0.46	0.64
Monocyte	0.72	0.071	0.40	0.07	0.34
NK cell	0.07	**0.037**	0.19	**0.02**	**0.0013**
Perivascular cell	0.59	0.81	**1.97e-06**	0.69	**0.013**
Proliferation T/NK	0.34	0.75	0.72	0.86	0.50

Table S23: **p-values of the Cox proportional hazard model** for all minor celltypes.

p-Values below 0.05 are highlighted in bold.

	all subtypes	HER2	LumA	LumB	TNBC
Arterial EC	-101.28	**43.41**	8.79	33.84	16.06
Breast basal cell	-8.48	14.53	8.91	**41.57**	4.17
Breast cancer specific luminal cell	**6.67**	**7.54**	0.43 (0.69)	-0.41	1.03
Breast cancer specific proliferation luminal cell	-0.11	3.92	-3.29	3.93	1.76
Capillary EC	4.42	2.76	1.54	-7.10	0.96
CD4 T	5.44	-5.71	**-9.56**	-1.71	-3.01
cDC2	-34.92	-34.29	-19.38	-4.35	**-22.78**
CFD fibroblast	3.21	-12.39	0.36	5.26	0.63
CXCL1/2/3 fibroblast	-37.48	-12.79	-18.85	17.57	-12.60
CXCL13 exhausted CD8 T	**-39.82**	-192.43	**-123.68**	-30.21	**-45.53**
GZMH CD8 T	3.40	-11.75	1.29	-7.33	-0.14
GZMK CD8 T	-2.68	-14.09	0.76	-6.35	-2.91
IgA plasma	-260.41	**-949.51**	179.55	18.35	-86.64
IgG plasma cell	-124.48	-369.38	3.53	-2.17	-77.70
INF responsed T	**80.28**	-5602.28	-60.85	20.41	33.68
Luminal progenitor	-1.17	-4.92	**-31.29**	-29.94	-7.14
Lymphatic EC	15.86	-3.06	-18.65	30.54	0.38
Macrophage	-1.67	**-21.08**	-3.37	2.36	-2.45
Mast cell	34.17	**-146.24**	-71.30	85.01	-14.90
Monocyte	7.20	-240.64	-66.99	-101.17	-20.30
mregDC	**-93.12**	22.12	-67.10	**-193.46**	**-83.75**
NK cell	-11.65	**-41.99**	-15.93	**-37.69**	**-21.05**
Other B cells	-12.39	-11.35	5.76	-8.51	-5.54
Other fibroblasts	-0.42	-15.39	2.25	**11.33**	2.24
pDC	45.06	23.47	-30.35	-91.80	-26.27
Pericyte	-8.50	44.12	**7.72**	15.86	**7.89**
Proliferation macrophage	31.82	-17.72	-53.75	32.55	13.24
Proliferation T/NK	-15.45	3.99	-6.23	1.09	-4.01
Smooth muscle cell	5.88	-14.89	11.25	-6.99	3.94
Tfh	30.05	-12.58	-75.13	**-160.34**	-62.06
TGM2 luminal cell	-11.76	8.05	-0.69	2.33	0.69
TNBC-specific epithelial cell	-2.55	-3.38	-19.74	**16.79**	-1.99
Treg	62.85	62.94	5.82	43.95	**27.90**
Venous EC	4.65	11.23	-20.41	**24.87**	-0.52

Table S24: **Coefficients of the Cox proportional hazard model** for all sub celltypes. Coefficients having a p-Value below 0.05 are highlighted in bold.

	all subtypes	HER2	LumA	LumB	TNBC
Arterial EC	0.20	**0.020**	0.61	0.16	0.18
Breast basal cell	0.49	0.60	0.20	**0.035**	0.46
Breast cancer specific luminal cell	**0.04**	**0.0066**	0.69	0.71	0.19
Breast cancer specific proliferation luminal cell	0.98	0.51	0.65	0.16	0.40
Capillary EC	0.34	0.78	0.46	0.23	0.59
CD4 T	0.25	0.49	**0.026**	0.79	0.31
cDC2	0.07	0.25	0.22	0.84	**0.025**
CFD fibroblast	0.48	0.11	0.89	0.21	0.74
CXCL1/2/3 fibroblast	0.13	0.10	0.41	0.29	0.10
CXCL13 exhausted CD8 T	**0.0036**	0.31	**0.036**	0.15	**0.00072**
GZMH CD8 T	0.41	0.20	0.61	0.25	0.95
GZMK CD8 T	0.61	0.26	0.84	0.21	0.29
IgA plasma	0.33	**0.037**	0.36	0.93	0.59
IgG plasma cell	0.24	0.06	0.97	0.98	0.18
INF responsed T	**0.00028**	0.23	0.33	0.72	0.11
Luminal progenitor	0.80	0.58	**0.0021**	0.26	0.11
Lymphatic EC	0.49	0.92	0.23	0.22	0.97
Macrophage	0.73	**0.047**	0.57	0.73	0.45
Mast cell	0.54	**0.024**	0.14	0.17	0.62
Monocyte	0.72	0.07	0.40	0.07	0.34
mregDC	**0.018**	0.72	0.40	**0.037**	**0.0037**
NK cell	0.073	**0.037**	0.19	**0.020**	**0.0013**
Other B cells	0.37	0.56	0.56	0.48	0.41
Other fibroblasts	0.94	0.17	0.71	**0.023**	0.47
pDC	0.57	0.86	0.13	0.20	0.48
Pericyte	0.79	0.15	**7.63e-16**	0.42	**1.5e-13**
Proliferation macrophage	0.14	0.53	0.15	0.13	0.33
Proliferation T/NK	0.34	0.75	0.72	0.86	0.50
Smooth muscle cell	0.48	0.45	0.15	0.42	0.38
Tfh	0.65	0.87	0.29	**0.03**	0.15
TGM2 luminal cell	0.48	0.35	0.92	0.82	0.88
TNBC-specific epithelial cell	0.51	0.68	0.27	**0.03**	0.55
Treg	0.093	0.29	0.77	0.18	**0.049**
Venous EC	0.86	0.53	0.13	**0.04**	0.95

Table S25: **p-values of the Cox proportional hazard model** for all sub celltypes. p-Values below 0.05 are highlighted in bold.

celltype	T>0N0M0	T>0N>0M>0	p
Endothelial cell	0.1085 ± 0.0493	0.1029 ± 0.0456	0.3048
Epithelial cell	0.4275 ± 0.1289	0.4192 ± 0.1342	0.7495
Fibroblast	0.0954 ± 0.0588	0.0937 ± 0.0602	0.6406
B cell	0.0268 ± 0.0155	0.0314 ± 0.0176	**0.0059**
NK cell	0.0334 ± 0.0136	0.0331 ± 0.0219	**0.0354**
Proliferation T NK	0.0115 ± 0.0133	0.0156 ± 0.0169	**0.0449**
T cell	0.1892 ± 0.0648	0.1910 ± 0.0765	0.8052
Myeloid cell	0.0711 ± 0.0354	0.0752 ± 0.0447	0.7183
Perivascular cell	0.0366 ± 0.0225	0.0378 ± 0.0277	0.9869
CD4 T cell	0.0763 ± 0.0325	0.0742 ± 0.0341	0.5799
CD8 T cell	0.1129 ± 0.0620	0.1167 ± 0.0717	0.9467
CD4 T	0.0625 ± 0.0304	0.0613 ± 0.0310	0.7341
Tfh	0.0029 ± 0.0037	0.0026 ± 0.0052	0.3138
Treg	0.0091 ± 0.0049	0.0090 ± 0.0054	0.6891
INF responsed T	0.0019 ± 0.0047	0.0014 ± 0.0036	0.8225
CXCL13 exhausted CD8 T	0.0049 ± 0.0116	0.0072 ± 0.0227	0.5145
GZMH CD8 T	0.0776 ± 0.0351	0.0781 ± 0.0370	0.8780
GZMK CD8 T	0.0305 ± 0.0291	0.0314 ± 0.0353	0.5732
other B cells	0.0241 ± 0.0137	0.0288 ± 0.0164	**0.0048**
IgA plasma	0.0007 ± 0.0009	0.0006 ± 0.0007	0.3838
IgG plasma cell	0.0020 ± 0.0025	0.0020 ± 0.0016	**0.0334**
Arterial EC	0.0047 ± 0.0075	0.0030 ± 0.0053	**0.0009**
Capillary EC	0.0843 ± 0.0506	0.0843 ± 0.0486	0.7470
Lymphatic EC	0.0091 ± 0.0069	0.0068 ± 0.0066	**0.0002**
Venous EC	0.0104 ± 0.0120	0.0087 ± 0.0090	0.2689
Luminal progenitor	0.0179 ± 0.0255	0.0264 ± 0.0397	0.1838
TGM2 luminal cell	0.0144 ± 0.0156	0.0165 ± 0.0173	0.3480
Breast cancer specific luminal cell	0.3145 ± 0.1435	0.2785 ± 0.1630	**0.0497**
Breast cancer specific proliferation luminal cell	0.0427 ± 0.0422	0.0489 ± 0.0478	0.4173
TNBC-specific epithelial cell	0.0231 ± 0.0412	0.0344 ± 0.0492	0.1013
Breast basal cell	0.0147 ± 0.0133	0.0145 ± 0.0122	0.8436
Pericyte	0.0084 ± 0.0081	0.0102 ± 0.0176	0.4703
Smooth muscle cell	0.0282 ± 0.0189	0.0277 ± 0.0189	0.6247
other fibroblasts	0.0228 ± 0.0251	0.0233 ± 0.0244	0.5831
CFD fibroblast	0.0669 ± 0.0497	0.0614 ± 0.0462	0.2686
CXCL1/2/3 fibroblast	0.0057 ± 0.0085	0.0090 ± 0.0219	**0.0038**
Dendritic cell	0.0172 ± 0.0093	0.0187 ± 0.0122	0.4760
Granulocyte	0.0071 ± 0.0034	0.0070 ± 0.0037	0.6150
Macrophages	0.0421 ± 0.0259	0.0447 ± 0.0324	0.9269
Monocyte	0.0047 ± 0.0086	0.0049 ± 0.0078	0.4500
Macrophage	0.0342 ± 0.0244	0.0359 ± 0.0302	0.7008
Proliferation macrophage	0.0079 ± 0.0075	0.0088 ± 0.0075	0.1769
cDC2	0.0140 ± 0.0082	0.0147 ± 0.0094	0.6507
mregDC	0.0019 ± 0.0036	0.0023 ± 0.0046	0.5732
pDC	0.0013 ± 0.0015	0.0018 ± 0.0022	**0.0387**

Table S26: **Kruskal Wallis Test for patients with only a primary tumor and patients having lymphnode involvement and metastatis.** Unknown Lymphnode involvement (NX) and metastasis levels (MX) are regarded as present. p-Values below 0.05 are highlighted in bold.

4 TCGA BayesPrism

celltype	All	Her2	LumA	LumB	TNBC
B cell	-9.4026	**-64.7146**	-14.1094	-0.4991	-13.2918
Endothelial cell	0.3126	0.2795	0.9111	-3.8546	0.7176
Epithelial cell	0.7337	**4.1682**	0.3881	-0.0016	0.9455
Fibroblast	0.2855	**-11.3337**	-0.6728	**5.5736**	1.8163
Myeloid cell	-1.3378	-7.4548	-2.7269	0.0410	0.0638
NK cell	-3.6520	-17.1880	-2.4874	-4.5490	**-6.5682**
Perivascular cell	1.5931	-3.7654	0.7040	7.4556	0.8324
Proliferation T/NK	-0.4208	-2.8571	0.4449	2.2205	-3.9174
T cell	-3.2064	3.7769	-0.1299	**-8.5666**	**6.9383**

Table S27: **Coefficients of the Cox proportional hazard model** for all BayesPrism major celltypes using the independent level approach. Coefficients having a p-Value below 0.05 are highlighted in bold.

celltype	All	Her2	LumA	LumB	TNBC
B cell	0.0739	**0.0247**	0.2727	0.9425	0.1384
Endothelial cell	0.8061	0.9434	0.5658	0.3066	0.8248
Epithelial cell	0.1839	**0.0005**	0.6267	0.9987	0.4969
Fibroblast	0.8546	**0.0471**	0.7792	**0.0250**	0.4165
Myeloid cell	0.3675	0.0740	0.2624	0.9878	0.9803
NK cell	0.0827	0.0957	0.3030	0.3337	**0.0256**
Perivascular cell	0.3947	0.5298	0.7756	0.1593	0.8000
Proliferation T/NK	0.7268	0.4276	0.8404	0.2113	0.1371
T cell	0.1294	0.4712	0.9559	**0.0303**	**0.0003**

Table S28: **p-values of the Cox proportional hazard model** for all BayesPrism major using the independent level approach. p-Values below 0.05 are highlighted in bold.

celltype	All	Her2	LumA	LumB	TNBC
B cell	-1.9036	1.9999	3.2306	-0.2849	-3.7519
Endothelial cell	1.5754	1.9921	1.2668	3.7207	1.3504
Epithelial cell	0.7662	**3.7228**	0.4464	-0.0510	0.6071
Fibroblast	0.3598	-9.9653	-0.9926	4.9110	3.1663
Myeloid cell	-2.0891	**-9.5628**	-3.7460	0.2339	0.1495
NK cell	-27.4687	-12.4174	-42.2026	-33.3553	9.3520
Perivascular cell	5.3841	**-26.2962**	**13.6436**	7.3365	-3.1559
Proliferation T/NK	-2.8569	-6.3572	3.4119	-7.9954	-1.9389
T cell	-3.6986	-7.8742	-1.3963	-5.7811	-3.1066

Table S29: **Coefficients of the Cox proportional hazard model** for all BayesPrism major celltypes using the summing approach. Coefficients having a p-Value below 0.05 are highlighted in bold.

celltype	All	Her2	LumA	LumB	TNBC
B cell	0.3045	0.6279	0.3980	0.9114	0.3697
Endothelial cell	0.1326	0.7043	0.4668	0.2881	0.3186
Epithelial cell	0.1827	**0.0019**	0.6045	0.9624	0.6660
Fibroblast	0.8046	0.1167	0.5965	0.0868	0.3753
Myeloid cell	0.1192	**0.0051**	0.0654	0.9264	0.9501
NK cell	0.0631	0.5745	0.0548	0.5503	0.4839
Perivascular cell	0.1444	**0.0266**	**0.0002**	0.4229	0.5474
Proliferation T/NK	0.2602	0.3242	0.6601	0.2851	0.6005
T cell	0.0179	0.0738	0.5718	0.0597	0.1789

Table S30: **p-values of the Cox proportional hazard model** for all BayesPrism major using the summing approach. p-Values below 0.05 are highlighted in bold.

celltype	All	Her2	LumA	LumB	TNBC
B cell	-8.3739	**-61.9155**	-12.5668	-0.5659	-12.8468
CD4 T cell	-0.9286	10.2179	0.9062	-2.4928	**10.1101**
CD8 T cell	**-5.8444**	-21.1047	-3.1365	-9.7985	-6.5525
Dendritic cell	0.9245	3.5107	2.2533	1.0173	-4.0091
Endothelial cell	0.4199	0.3037	0.9572	-4.1710	0.9648
Epithelial cell	0.8298	**3.8595**	0.4985	-0.1420	0.6830
Fibroblast	0.2646	**-10.9716**	-0.3389	**5.1962**	0.8777
Granulocyte	-0.5295	2.0843	-2.4196	**8.1836**	**4.0152**
Macrophage	0.0882	-3.8284	-3.1627	1.9051	2.5938
Monocyte	-2.3031	-5.6797	-1.7471	-3.2309	-1.6619
NK cell	0.9951	-19.8187	-1.8088	-1.5835	**12.1541**
Perivascular cell	2.9622	-7.5198	6.8891	2.3795	-1.0588
Proliferation T/NK	-0.9049	-0.9283	1.6681	0.9201	-3.0189

Table S31: **Coefficients of the Cox proportional hazard model** for all BayesPrism minor celltypes using the independent level approach. Coefficient having a p-Value below 0.05 are highlighted in bold.

celltype	All	Her2	LumA	LumB	TNBC
B cell	0.0704	**0.0279**	0.2695	0.9265	0.1352
CD4 T cell	0.7032	0.1509	0.7540	0.5626	**0.0308**
CD8 T cell	**0.0309**	0.0905	0.3650	0.0757	0.0510
Dendritic cell	0.5993	0.5070	0.2801	0.8156	0.2999
Endothelial cell	0.7380	0.9417	0.5430	0.3175	0.7541
Epithelial cell	0.1484	**0.0010**	0.5371	0.8983	0.6530
Fibroblast	0.8660	**0.0427**	0.8901	**0.0315**	0.7414
Granulocyte	0.6643	0.6467	0.1408	**0.0037**	**0.0115**
Macrophage	0.9599	0.5234	0.2603	0.6164	0.3963
Monocyte	0.1589	0.2579	0.5375	0.2737	0.4894
NK cell	0.7677	0.2156	0.6223	0.8377	**0.0037**
Perivascular cell	0.2330	0.3811	0.0729	0.6848	0.7756
Proliferation T/NK	0.5034	0.8023	0.4889	0.6604	0.2718

Table S32: **p-values of the Cox proportional hazard model** for all BayesPrism minor celltypes using the independent level approach. p-Values below 0.05 are highlighted in bold.

celltype	All	Her2	LumA	LumB	TNBC
B cell	-1.9036	1.9999	3.2306	-0.2849	-3.7519
CD4 T cell	1.5606	3.0184	0.2464	0.0122	7.1549
CD8 T cell	**-4.5455**	-12.5079	-1.3808	-5.4063	**-6.7251**
Dendritic cell	-4.2210	-18.3559	-2.4806	-7.2820	-6.6139
Endothelial cell	1.5754	1.9921	1.2668	3.7207	1.3504
Epithelial cell	0.7662	**3.7228**	0.4464	-0.0510	0.6071
Fibroblast	0.3598	-9.9653	-0.9926	4.9110	3.1663
Granulocyte	-0.6795	-18.4340	-4.3766	**9.6800**	7.9100
Macrophage	1.3040	-1.7956	-3.1875	4.9482	3.9999
Monocyte	**-5.9500**	-5.5658	0.6082	**-10.2672**	-4.0897
NK cell	-27.4687	-12.4174	-42.2026	-33.3553	9.3520
Perivascular cell	5.3841	**-26.2962**	**13.6436**	7.3365	-3.1559
Proliferation T/NK	-2.8569	-6.3572	3.4119	-7.9954	-1.9389

Table S33: **Coefficients of the Cox proportional hazard model** for all BayesPrism minor celltypes using the summing approach. Coefficients having a p-Value below 0.05 are highlighted in bold.

celltype	All	Her2	LumA	LumB	TNBC
B cell	0.3045	0.6279	0.3980	0.9114	0.3697
CD4 T cell	0.4935	0.5952	0.9372	0.9974	0.0817
CD8 T cell	**0.0114**	0.1216	0.5559	0.0874	**0.0434**
Dendritic cell	0.1202	0.1479	0.4813	0.2911	0.3735
Endothelial cell	0.1326	0.7043	0.4668	0.2881	0.3186
Epithelial cell	0.1827	**0.0019**	0.6045	0.9624	0.6660
Fibroblast	0.8046	0.1167	0.5965	0.0868	0.3753
Granulocyte	0.8139	0.4482	0.2337	**0.0009**	0.3527
Macrophage	0.3569	0.6384	0.2017	0.0962	0.1079
Monocyte	**0.0238**	0.3585	0.8823	**0.0364**	0.2143
NK cell	0.0631	0.5745	0.0548	0.5503	0.4839
Perivascular cell	0.1444	**0.0266**	**0.0002**	0.4229	0.5474
Proliferation T/NK	0.2602	0.3242	0.6601	0.2851	0.6005

Table S34: **p-values of the Cox proportional hazard model** for all BayesPrism minor celltypes using the summing approach. p-Values below 0.05 are highlighted in bold.

celltype	All	Her2	LumA	LumB	TNBC
Arterial EC	**5.4162**	5.2465	4.1931	**9.2239**	2.3352
Breast basal cell	3.7439	-87.3063	-6.2313	28.6982	4.7195
Breast cancer specific luminal cell	0.6825	1.0364	1.5076	0.1862	2.4618
Breast cancer specific proliferation luminal cell	0.8133	1.0754	-0.8467	-0.3814	-1.1846
Capillary EC	-0.0650	3.8104	3.2469	**-17.3285**	0.6174
CD4 T	4.5263	**18.6437**	3.0040	3.3209	4.7720
cDC2	-2.4607	-6.7605	0.1389	-21.1442	-11.7422
CFD fibroblast	-0.5641	-6.7215	-1.8336	0.5398	4.5876
CXCL1/2/3 fibroblast	-17.4739	**-346.9702**	-0.4755	11.7649	-6.4773
CXCL13 exhausted CD8 T	**-19.1281**	-29.0025	-19.9742	-11.1058	**-21.5443**
GZMH CD8 T	-9.6149	-57.0463	-16.7306	1.5366	-16.8151
GZMK CD8 T	-1.6261	-3.2986	0.1871	-11.2952	2.9192
IgA plasma	-10.9764	**-898.1144**	-6.7250	18.6267	-56.9004
IgG plasma cell	-17.1157	**-1833.3516**	-38.0984	-3.1572	-86.8569
INF responsed T	-2.2890	-8.7228	1.6581	-2.0449	-3.1449
Luminal progenitor	-1.4996	3.7748	-4.0715	-14.2509	-0.7606
Lymphatic EC	-0.3698	-8.7084	-6.1128	14.7250	0.8503
Macrophage	-2.5452	-3.6832	-5.5721	-2.3108	-7.0242
Mast cell	-0.6795	-18.4340	-4.3766	**9.6800**	7.9100
Monocyte	**-5.9500**	-5.5658	0.6082	-10.2672	-4.0897
mregDC	-5.8331	**-88.7533**	-10.3124	3.8403	**-32.9024**
NK cell	-27.4687	-12.4174	-42.2026	-33.3553	9.3520
Other B cells	0.3459	2.9760	5.6792	-0.2962	-3.2714
Other fibroblasts	3.2253	-20.0772	2.9833	7.5250	3.3702
pDC	-1.0041	-124.1435	-1.0385	-58.3741	**14.9171**
Pericyte	5.4178	**-475.7124**	**12.4094**	**32.3900**	-13.7003
Proliferation macrophage	2.5757	0.4828	-1.5789	**8.0697**	**5.4180**
Proliferation T/NK	-2.8569	-6.3572	3.4119	-7.9954	-1.9389
Smooth muscle cell	4.2939	-13.4208	10.5311	-0.8988	0.5776
Tfh	-26.8988	-410.5044	**-28.0473**	-18.3484	**22.7583**
TGM2 luminal cell	-1.1357	**1.7665**	-2.1383	-5.5970	3.3998
TNBC-specific epithelial cell	0.2846	-6.9020	-1.7023	3.7820	0.7186
Treg	4.0164	-13.8156	-2.0061	-18.7055	**9.9284**
Venous EC	-27.2343	-260.8903	-38.0431	-12.4781	-6.1618

Table S35: **Coefficients of the Cox proportional hazard model** for all BayesPrism sub celltypes. Coefficients having a p-Value below 0.05 are highlighted in bold.

celltype	All	Her2	LumA	LumB	TNBC
Arterial EC	**0.0029**	0.3336	0.0712	**0.0306**	0.5715
Breast basal cell	0.5419	0.1683	0.5932	0.3493	0.2689
Breast cancer specific luminal cell	0.2741	0.5504	0.0684	0.8461	0.2304
Breast cancer specific proliferation luminal cell	0.2680	0.4422	0.4948	0.7792	0.5104
Capillary EC	0.9835	0.7064	0.3868	**0.0486**	0.9259
CD4 T	0.0895	**0.0084**	0.3823	0.4356	0.4661
cDC2	0.4872	0.4701	0.9741	0.1168	0.3535
CFD fibroblast	0.7292	0.4267	0.3273	0.9110	0.3183
CXCL1/2/3 fibroblast	0.1784	**0.0212**	0.9576	0.2719	0.6235
CXCL13 exhausted CD8 T	**0.0044**	0.2192	0.2404	0.2046	**0.0275**
GZMH CD8 T	0.1185	0.3006	0.1844	0.8681	0.0814
GZMK CD8 T	0.4617	0.6509	0.9376	0.0521	0.5139
IgA plasma	0.6757	**0.0175**	0.8250	0.3331	0.4658
IgG plasma cell	0.0643	**0.0179**	0.1977	0.8263	0.1965
INF responsed T	0.4629	0.2893	0.7021	0.7717	0.5410
Luminal progenitor	0.4763	0.0801	0.3770	0.2203	0.7649
Lymphatic EC	0.8214	0.5054	0.2375	0.1433	0.5403
Macrophage	0.2691	0.4707	0.1653	0.6555	0.1062
Mast cell	0.8139	0.4482	0.2337	**0.0009**	0.3527
Monocyte	**0.0238**	0.3585	0.8823	0.0364	0.2143
mregDC	0.1298	**0.0189**	0.1699	0.5750	**0.0240**
NK cell	0.0631	0.5745	0.0548	0.5503	0.4839
Other B cells	0.8612	0.4538	0.1313	0.9141	0.4124
Other fibroblasts	0.1675	0.1260	0.3846	0.0137	0.5880
pDC	0.9170	0.0656	0.9113	0.1102	**0.0001**
Pericyte	0.2905	**0.0063**	**3.54e-17**	**1.39e-10**	0.3103
Proliferation macrophage	0.0955	0.9020	0.6216	**0.0117**	**0.0208**
Proliferation T/NK	0.2602	0.3242	0.6601	0.2851	0.6005
Smooth muscle cell	0.3048	0.2641	0.1356	0.9303	0.9196
Tfh	0.0388	0.1696	**0.0301**	0.6884	**0.0019**
TGM2 luminal cell	0.4735	**0.0110**	0.3295	0.1404	0.5081
TNBC-specific epithelial cell	0.8662	0.2843	0.6119	0.0617	0.7235
Treg	0.4820	0.3455	0.9030	0.1762	**0.0257**
Venous EC	0.0981	0.0800	0.2575	0.4400	0.8208

Table S36: **p-values of the Cox proportional hazard model** for all BayesPrism sub celltypes. p-Values below 0.05 are highlighted in bold.

5 TCGA CIBERSORTx

celltype	All	Her2	LumA	LumB	TNBC
B cell	-30.5645	**-146.5029**	-69.2071	-0.5262	-26.7897
Endothelial cell	-2.6442	1.6921	-3.4979	-4.3697	-1.6703
Epithelial cell	1.0285	**4.5129**	0.4756	0.6439	1.4728
Fibroblast	1.6019	**-15.4705**	1.4337	**7.3956**	0.7297
Myeloid cell	-1.5213	**-11.9837**	-0.1064	0.4051	-1.5463
NK cell	0.6909	-8.2995	1.0484	0.1390	1.7483
Perivascular cell	2.8731	6.6161	**6.8196**	0.8350	-2.7251
Proliferation T/NK	-0.9843	-3.7996	2.3543	2.1615	-4.3189
T cell	**-1.1227**	0.1931	-1.4955	-2.1026	0.0227

Table S37: **Coefficients of the Cox proportional hazard model** for all CIBERSORTx major celltypes using the independent level approach. Coefficients having a p-Value below 0.05 are highlighted in bold.

celltype	All	Her2	LumA	LumB	TNBC
B cell	0.0995	**0.0232**	0.1910	0.9819	0.2085
Endothelial cell	0.1517	0.7382	0.2851	0.1966	0.6445
Epithelial cell	0.0809	**0.0094**	0.6289	0.4734	0.2585
Fibroblast	0.4700	**0.0395**	0.6477	**0.0126**	0.9044
Myeloid cell	0.3591	**0.0093**	0.9717	0.8962	0.5512
NK cell	0.4777	0.1249	0.2863	0.9519	0.3077
Perivascular cell	0.0581	0.1201	**0.0153**	0.8122	0.3433
Proliferation T/NK	0.4707	0.2271	0.5752	0.1639	0.0956
T cell	**0.0387**	0.9082	0.0519	0.0639	0.9861

Table S38: **p-values of the Cox proportional hazard model** for all CIBERSORTx major using the independent level approach. p-Value below 0.05 are highlighted in bold.

celltype	All	Her2	LumA	LumB	TNBC
B cell	1.0080	-2.0793	5.1925	0.1355	-2.1486
Endothelial cell	-0.0855	4.6375	-0.7636	-0.2855	-1.4472
Epithelial cell	**1.8676**	**9.0811**	**2.6149**	-1.0672	0.5206
Fibroblast	1.7907	-8.1620	0.5728	6.1803	6.2750
Myeloid cell	-0.2141	-2.8775	1.0888	-2.2058	0.8939
NK cell	-3.8545	3.6064	-7.8695	1.8717	-9.0168
Perivascular cell	4.5343	16.9347	6.8825	5.9400	-1.2718
Proliferation T/NK	-1.1541	-16.4496	0.5130	6.9417	-3.8825
T cell	**-2.0098**	-4.7869	**-3.1352**	0.3225	-0.1816

Table S39: **Coefficients of the Cox proportional hazard model** for all CIBERSORTx major celltypes using the summing approach. Coefficients having a p-Value below 0.05 are highlighted in bold.

celltype	All	Her2	LumA	LumB	TNBC
B cell	0.5844	0.8292	0.1166	0.9632	0.5809
Endothelial cell	0.9710	0.4251	0.8425	0.9482	0.7853
Epithelial cell	**0.0119**	**0.0062**	**0.0063**	0.4812	0.8021
Fibroblast	0.3978	0.2555	0.8413	0.1571	0.2594
Myeloid cell	0.8841	0.3925	0.6458	0.4855	0.7607
NK cell	0.3233	0.7464	0.1184	0.5226	0.4657
Perivascular cell	0.3724	0.3310	0.4569	0.6431	0.8615
Proliferation T/NK	0.7165	0.0536	0.9312	0.1564	0.5568
T cell	**0.0026**	0.1652	**0.0004**	0.7976	0.9257

Table S40: **p-values of the Cox proportional hazard model** for all CIBERSORTx major celltypes using the summing approach. p-Values below 0.05 are highlighted in bold.

celltype	All	Her2	LumA	LumB	TNBC
B cell	-32.2873	**-189.8166**	-93.0329	5.6408	-24.5717
CD4 T cell	-1.0070	0.3066	**-2.5996**	0.6155	0.2983
CD8 T cell	-1.3177	-4.5065	0.5466	-2.2159	-1.9327
Dendritic cell	0.5448	1.0380	**3.8735**	-3.9657	-2.8631
Endothelial cell	-1.4733	2.6262	-3.8111	-2.0302	0.1074
Epithelial cell	1.0693	**4.6741**	0.3027	0.9482	1.6790
Fibroblast	1.3533	**-17.5426**	0.7355	**7.7326**	4.2966
Granulocyte	1.5295	1.4963	**2.8595**	-3.1937	2.4779
Macrophage	1.4289	1.0777	2.5402	3.3707	-2.1966
Monocyte	-1.2728	-2.2530	-6.8876	-1.3487	1.7960
NK cell	0.8231	-15.2769	1.6350	-1.0131	2.8166
Perivascular cell	4.1272	9.2994	**8.9826**	3.5901	-3.4788
Proliferation T/NK	-0.0126	-2.8164	3.3996	**5.2199**	-5.1262

Table S41: **Coefficients of the Cox proportional hazard model** for all CIBERSORTx minor celltypes using the independent level approach. Coefficients having a p-Value below 0.05 are highlighted in bold.

celltype	All	Her2	LumA	LumB	TNBC
B cell	0.1088	**0.0412**	0.2728	0.8445	0.2671
CD4 T cell	0.0512	0.8381	**0.0008**	0.6018	0.7846
CD8 T cell	0.1467	0.1065	0.6989	0.3187	0.1859
Dendritic cell	0.6034	0.7329	**0.0076**	0.1005	0.2675
Endothelial cell	0.4950	0.6503	0.3318	0.6012	0.9799
Epithelial cell	0.0992	**0.0073**	0.7784	0.3433	0.2646
Fibroblast	0.5868	**0.0262**	0.8333	**0.0146**	0.5751
Granulocyte	0.0901	0.6197	**0.0124**	0.1269	0.3543
Macrophage	0.3792	0.8158	0.3448	0.3322	0.5061
Monocyte	0.3825	0.5623	0.0954	0.7077	0.3708
NK cell	0.5934	0.0901	0.3375	0.7242	0.4534
Perivascular cell	0.0905	0.2123	**0.0336**	0.5475	0.4500
Proliferation T/NK	0.9942	0.4278	0.4970	**0.0304**	0.1199

Table S42: **p-values of the Cox proportional hazard model** for all CIBERSORTx minor celltypes using the independent level approach. p-Values below 0.05 are highlighted in bold.

celltype	All	Her2	LumA	LumB	TNBC
B cell	1.0080	-2.0793	5.1925	0.1355	-2.1486
CD4 T cell	**-1.6920**	-0.9971	**-3.0518**	0.5386	1.0174
CD8 T cell	-2.1995	-6.3099	-4.9113	0.1535	-1.1925
Dendritic cell	0.6555	-0.7076	**5.4189**	-4.9126	-5.3231
Endothelial cell	-0.0855	4.6375	-0.7636	-0.2855	-1.4472
Epithelial cell	**1.8676**	**9.0811**	**2.6149**	-1.0672	0.5206
Fibroblast	1.7907	-8.1620	0.5728	6.1803	6.2750
Granulocyte	0.1916	3.2861	4.9065	**-19.8203**	14.1856
Macrophage	1.5205	-2.6807	-3.4299	7.8862	6.3007
Monocyte	-6.1145	-7.9232	-17.0104	5.5343	-0.7546
NK cell	-3.8545	3.6064	-7.8695	1.8717	-9.0168
Perivascular cell	4.5343	16.9347	6.8825	5.9400	-1.2718
Proliferation T/NK	-1.1541	-16.4496	0.5130	6.9417	-3.8825

Table S43: **Coefficients of the Cox proportional hazard model** for all CIBERSORTx minor celltypes using the summing approach. Coefficients having a p-Value below 0.05 are highlighted in bold.

celltype	All	Her2	LumA	LumB	TNBC
B cell	0.5844	0.8292	0.1166	0.9632	0.5809
CD4 T cell	**0.0353**	0.7088	**0.0022**	0.7984	0.6559
CD8 T cell	0.0718	0.1099	0.0912	0.9294	0.5430
Dendritic cell	0.7099	0.8897	**0.0206**	0.2330	0.2095
Endothelial cell	0.9710	0.4251	0.8425	0.9482	0.7853
Epithelial cell	**0.0119**	**0.0062**	**0.0063**	0.4812	0.8021
Fibroblast	0.3978	0.2555	0.8413	0.1571	0.2594
Granulocyte	0.9559	0.8028	0.2402	**0.0174**	0.1311
Macrophage	0.5117	0.5972	0.3369	0.1754	0.1537
Monocyte	0.2175	0.4714	0.1159	0.5193	0.9240
NK cell	0.3233	0.7464	0.1184	0.5226	0.4657
Perivascular cell	0.3724	0.3310	0.4569	0.6431	0.8615
Proliferation T/NK	0.7165	0.0536	0.9312	0.1564	0.5568

Table S44: **p-values of the Cox proportional hazard model** for all CIBERSORTx minor celltypes using the summing approach. p-Values below 0.05 are highlighted in bold.

celltype	All	Her2	LumA	LumB	TNBC
Arterial EC	-1.8795	4.8244	**-11.3987**	4.5721	1.4638
Breast basal cell	12.3984	-23.7961	**29.9111**	-20.2442	10.6996
Breast cancer specific luminal cell	**1.7314**	1.9369	**3.0999**	-1.7892	3.7397
Breast cancer specific proliferation luminal cell	-1.0762	1.1540	-5.4654	1.0490	-1.9234
Capillary EC	3.2846	0.8178	**10.2084**	-3.8459	-3.5956
CD4 T	-1.1449	0.4376	**-2.5480**	0.5727	0.8523
cDC2	0.2481	-0.8170	3.4038	-8.9386	-2.2260
CFD fibroblast	1.6259	-7.3083	0.5129	5.4173	6.2812
CXCL1/2/3 fibroblast	5.4496	319.1339	-1233.0197	-393.3546	23.0349
CXCL13 exhausted CD8 T	**-5.4256**	-1.1223	**-9.4474**	-1.8589	-4.4100
GZMH CD8 T	1.0818	-8.0858	5.0644	-0.0695	1.8194
GZMK CD8 T	0.7263	-3.6779	1.3755	4.1805	-1.1439
IgA plasma	-43.5743	-636.3439	-1.6715	135.3391	-108.8096
IgG plasma cell	-68.3065	-517.2756	-128.7192	87.7805	-54.4150
INF responsed T	-4.6302	-6.0812	-20.0574	6.2810	-2.4731
Luminal progenitor	-4.6509	2.5084	**-26.4033**	2.7194	1.1480
Lymphatic EC	-2.7765	10.9447	-1.9077	-14.3284	0.4273
Macrophage	-5.4976	-8.6591	-5.9630	-2.6794	-5.9791
Mast cell	0.1916	3.2861	4.9065	**-19.8203**	14.1856
Monocyte	-6.1145	-7.9232	-17.0104	5.5343	-0.7546
mregDC	-2.7398	-24.2343	**25.9567**	3.8258	-31.2626
NK cell	-3.8545	3.6064	-7.8695	1.8717	-9.0168
Other B cells	1.1911	0.5849	5.1823	-0.0505	-1.6809
Other fibroblasts	7.7793	-68.2413	4.7564	**22.6254**	2.7237
pDC	3.2878	9.6755	11.3761	-7.9035	-11.9171
Pericyte	26.9437	-148.0320	34.9562	-1.3019	**47.2196**
Proliferation macrophage	3.6172	0.7859	-1.2147	8.1132	**8.3928**
Proliferation T/NK	-1.1541	-16.4496	0.5130	6.9417	-3.8825
Smooth muscle cell	2.9662	18.7512	5.3912	6.4947	-4.8754
Tfh	-4.1208	-1.5094	-10.0390	-0.3073	7.5492
TGM2 luminal cell	**3.5755**	7.2149	**5.5518**	-1.9633	4.5180
TNBC-specific epithelial cell	0.2150	-2.9131	-8.6400	**25.0908**	-1.7894
Treg	0.7153	-3.9913	5.5743	-1.2201	-1.4882
Venous EC	**-340.1598**	**-3996.5278**	**-337.3606**	**-15122.9634**	-172.8930

Table S45: **Coefficients of the Cox proportional hazard model** for all CIBERSORTx sub celltypes. Coefficients having a p-Value below 0.05 are highlighted in bold.

celltype	All	Her2	LumA	LumB	TNBC
Arterial EC	0.4884	0.5562	**0.0330**	0.3160	0.7819
Breast basal cell	0.2988	0.7328	**0.0343**	0.5385	0.6593
Breast cancer specific luminal cell	**0.0233**	0.4938	**0.0018**	0.2015	0.4735
Breast cancer specific proliferation luminal cell	0.3606	0.6998	0.1289	0.5382	0.3967
Capillary EC	0.2047	0.9239	**0.0068**	0.5605	0.5613
CD4 T	0.0877	0.8183	**0.0032**	0.7307	0.6201
cDC2	0.9180	0.8963	0.2662	0.3859	0.6368
CFD fibroblast	0.4579	0.3482	0.8587	0.2740	0.2886
CXCL1/2/3 fibroblast	0.9409	0.2635	0.1659	0.0516	0.7332
CXCL13 exhausted CD8 T	**0.0005**	0.8193	**0.0003**	0.4415	0.1013
GZMH CD8 T	0.4969	0.1216	0.1170	0.9823	0.4493
GZMK CD8 T	0.7081	0.5264	0.6606	0.2658	0.7907
IgA plasma	0.6360	0.1350	0.9908	0.0722	0.5041
IgG plasma cell	0.1895	0.0835	0.2613	0.1662	0.3893
INF responsed T	0.4990	0.6912	0.2407	0.5983	0.8149
Luminal progenitor	0.2194	0.6952	**0.0239**	0.9209	0.8098
Lymphatic EC	0.6161	0.4141	0.8217	0.2139	0.9722
Macrophage	0.1143	0.4110	0.3204	0.7003	0.3593
Mast cell	0.9559	0.8028	0.2402	**0.0174**	0.1311
Monocyte	0.2175	0.4714	0.1159	0.5193	0.9240
mregDC	0.7002	0.1857	**0.0416**	0.7590	0.1205
NK cell	0.3233	0.7464	0.1184	0.5226	0.4657
Other B cells	0.5103	0.9418	0.1110	0.9865	0.6578
Other fibroblasts	0.3736	0.0933	0.7346	**0.0497**	0.8902
pDC	0.3863	0.3760	**0.0225**	0.3394	0.2743
Pericyte	0.1720	0.1010	0.3239	0.9739	**0.0492**
Proliferation macrophage	0.0904	0.8588	0.7163	0.1056	**0.0401**
Proliferation T/NK	0.7165	0.0536	0.9312	0.1564	0.5568
Smooth muscle cell	0.5655	0.2510	0.5590	0.6313	0.5348
Tfh	0.5250	0.8774	0.4648	0.9759	0.6981
TGM2 luminal cell	**0.0073**	0.0507	**0.0019**	0.3905	0.1619
TNBC-specific epithelial cell	0.9398	0.6960	0.2683	**0.0015**	0.6349
Treg	0.7038	0.4778	0.0611	0.7550	0.6987
Venous EC	**0.0012**	**3.05e-78**	**0.0119**	**3.85e-44**	0.1374

Table S46: **p-values of the Cox proportional hazard model** for all CIBERSORTx sub celltypes. p-Value below 0.05 are highlighted in bold.

6 TCGA Music

celltype	All	Her2	LumA	LumB	TNBC
B cell	-15.0262	**-63.7101**	-22.6750	-1.0627	-13.9066
Endothelial cell	**1.9767**	2.3415	**4.3318**	0.4540	-0.9170
Epithelial cell	0.6277	**3.0902**	-0.2800	0.8966	0.9763
Fibroblast	1.2098	-13.1964	-0.2189	**8.0090**	3.8823
Myeloid cell	-1.5676	-11.1384	-2.2578	0.6989	-0.3499
NK cell	0.2327	-3.4043	**2.9858**	**-6.0185**	0.9393
Perivascular cell	-0.6466	-16.5175	-0.8870	**14.3707**	-6.8713
Proliferation T/NK	-1.0868	-0.3015	-5.1070	4.2334	-4.5293
T cell	**-1.0991**	-1.5280	**-1.8116**	-0.9978	0.1417

Table S47: **Coefficients of the Cox proportional hazard model** for all Music major celltypes using the independent level approach. Coefficients having a p-Value below 0.05 are highlighted in bold.

celltype	All	Her2	LumA	LumB	TNBC
B cell	0.0888	**0.0369**	0.4156	0.9356	0.1489
Endothelial cell	**0.0348**	0.3618	**0.0031**	0.8543	0.6503
Epithelial cell	0.2414	**0.0079**	0.7553	0.3210	0.3944
Fibroblast	0.6291	0.1174	0.9538	**0.0322**	0.4613
Myeloid cell	0.3864	0.0506	0.5172	0.8478	0.8930
NK cell	0.8600	0.4966	**0.0223**	**0.0360**	0.8201
Perivascular cell	0.8465	0.3967	0.8728	**0.0460**	0.1792
Proliferation T/NK	0.5484	0.9313	0.5171	0.1671	0.1405
T cell	**0.0384**	0.3432	**0.0358**	0.3463	0.8824

Table S48: **p-values of the Cox proportional hazard model** for all Music major using the independent level approach. p-Values below 0.05 are highlighted in bold.

celltype	All	Her2	LumA	LumB	TNBC
B cell	**-12.5895**	-16.1119	-25.1186	-2.9773	-19.5719
Endothelial cell	**2.3719**	3.2944	**5.8514**	0.8567	-1.3676
Epithelial cell	0.8607	**4.4313**	0.2812	0.9580	0.6239
Fibroblast	1.5644	-9.5816	0.7515	5.5917	4.6401
Myeloid cell	-2.9100	-10.3902	-2.4607	-4.7918	-0.6645
NK cell	-6.1467	-17.8020	-11.6630	-3.2864	1.8471
Perivascular cell	0.9164	-15.2761	4.8041	10.0359	-8.4703
Proliferation T/NK	-3.0635	-68.8427	**48.0098**	**20.3554**	-8.0451
T cell	**-1.4617**	-2.3480	-1.9764	-2.3099	0.9871

Table S49: **Coefficients of the Cox proportional hazard model** for all Music major celltypes using the summing approach. Coefficients having a p-Value below 0.05 are highlighted in bold.

celltype	All	Her2	LumA	LumB	TNBC
B cell	**0.0144**	0.4730	0.0642	0.5225	0.0758
Endothelial cell	**0.0460**	0.2928	**0.0043**	0.7963	0.5292
Epithelial cell	0.1389	**0.0019**	0.7720	0.3116	0.6049
Fibroblast	0.4380	0.1682	0.7873	0.1227	0.2909
Myeloid cell	0.0646	0.0502	0.3058	0.1810	0.8030
NK cell	0.3874	0.4007	0.4262	0.8355	0.8505
Perivascular cell	0.8233	0.3295	0.5356	0.1758	0.1912
Proliferation T/NK	0.5410	0.0819	**0.0017**	**0.0282**	0.2274
T cell	**0.0365**	0.1958	0.0953	0.0856	0.4615

Table S50: **p-values of the Cox proportional hazard model** for all Music major using the summing approach. p-Values below 0.05 are highlighted in bold.

celltype	All	Her2	LumA	LumB	TNBC
B cell	-16.0772	**-61.3916**	-26.9755	-1.4038	-14.7935
CD4 T cell	-0.6139	0.5235	**-2.2186**	1.6237	0.1060
CD8 T cell	-1.8955	**-6.9536**	0.6447	**-5.9355**	-0.0883
Dendritic cell	**-7.4797**	-3.7065	-6.2841	-13.7109	-7.9955
Endothelial cell	**2.0398**	2.4965	**4.6267**	0.7871	-1.6314
Epithelial cell	0.6110	**3.1671**	-0.3956	1.0695	0.9592
Fibroblast	1.2143	-13.9620	-0.3527	**8.0531**	3.6913
Granulocyte	1.3877	7.1218	1.6861	-3.5309	**4.8230**
Macrophage	1.1738	-13.7834	-0.3240	7.0647	3.2809
Monocyte	-2.4943	-4.8490	-10.0256	1.1635	-1.8016
NK cell	0.1886	-2.7974	**7.3922**	-11.1561	2.2610
Perivascular cell	-1.0837	-18.3956	-2.2306	**16.4095**	-4.9615
Proliferation T/NK	-0.7848	0.1810	-7.3386	5.5795	-4.2387

Table S51: **Coefficients of the Cox proportional hazard model** for all Music minor celltypes using the independent level approach. Coefficients having a p-Value below 0.05 are highlighted in bold.

celltype	All	Her2	LumA	LumB	TNBC
B cell	0.0765	**0.0441**	0.3793	0.9171	0.1355
CD4 T cell	0.3472	0.7914	**0.0279**	0.2401	0.9291
CD8 T cell	0.0703	**0.0154**	0.6554	**0.0099**	0.9632
Dendritic cell	**0.0039**	0.6178	0.0962	0.0899	0.0777
Endothelial cell	**0.0372**	0.3354	**0.0033**	0.7549	0.3900
Epithelial cell	0.2502	**0.0059**	0.6532	0.2483	0.3980
Fibroblast	0.6237	0.1058	0.9252	**0.0280**	0.4646
Granulocyte	0.2238	0.3904	0.1585	0.3119	**0.0047**
Macrophage	0.6550	0.1591	0.9511	0.2716	0.3026
Monocyte	0.4032	0.5192	0.2728	0.8331	0.6403
NK cell	0.9471	0.7431	**0.0255**	0.0534	0.7560
Perivascular cell	0.7690	0.3023	0.7288	**0.0376**	0.3821
Proliferation T/NK	0.6820	0.9595	0.4286	0.1030	0.1972

Table S52: **p-values of the Cox proportional hazard model** for all Music minor celltypes using the independent level approach. p-Values below 0.05 are highlighted in bold.

celltype	All	Her2	LumA	LumB	TNBC
B cell	**-12.5895**	-16.1119	-25.1186	-2.9773	-19.5719
CD4 T cell	-0.5987	-0.2908	**-2.3221**	0.8177	2.2329
CD8 T cell	-1.1408	-3.2134	2.2584	**-4.4874**	-2.0370
Dendritic cell	-3.8439	-2.4944	-2.8749	-7.1366	-3.5879
Endothelial cell	**2.3719**	3.2944	**5.8514**	0.8567	-1.3676
Epithelial cell	0.8607	**4.4313**	0.2812	0.9580	0.6239
Fibroblast	1.5644	-9.5816	0.7515	5.5917	4.6401
Granulocyte	-0.2175	1.1490	-1.3997	-5.1487	**16.5062**
Macrophage	3.0362	-10.1366	1.2223	6.8377	6.6964
Monocyte	**-9.4069**	-12.5804	-4.9298	-9.4491	-9.3925
NK cell	-6.1467	-17.8020	-11.6630	-3.2864	1.8471
Perivascular cell	0.9164	-15.2761	4.8041	10.0359	-8.4703
Proliferation T/NK	-3.0635	-68.8427	**48.0098**	**20.3554**	-8.0451

Table S53: **Coefficients of the Cox proportional hazard model** for all Music minor celltypes using the summing approach. Coefficients having a p-Value below 0.05 are highlighted in bold.

celltype	All	Her2	LumA	LumB	TNBC
B cell	**0.0144**	0.4730	0.0642	0.5225	0.0758
CD4 T cell	0.3480	0.8603	**0.0166**	0.5014	0.1202
CD8 T cell	0.2655	0.2826	0.1091	**0.0171**	0.3337
Dendritic cell	0.0997	0.7242	0.3909	0.1614	0.4616
Endothelial cell	**0.0460**	0.2928	**0.0043**	0.7963	0.5292
Epithelial cell	0.1389	**0.0019**	0.7720	0.3116	0.6049
Fibroblast	0.4380	0.1682	0.7873	0.1227	0.2909
Granulocyte	0.9303	0.9743	0.5860	0.5081	**0.0006**
Macrophage	0.2898	0.2207	0.8081	0.3069	0.1437
Monocyte	**0.0308**	0.3181	0.7094	0.3937	0.0843
NK cell	0.3874	0.4007	0.4262	0.8355	0.8505
Perivascular cell	0.8233	0.3295	0.5356	0.1758	0.1912
Proliferation T/NK	0.5410	0.0819	**0.0017**	**0.0282**	0.2274

Table S54: **p-values of the Cox proportional hazard model** for all Music minor celltypes using the summing approach. p-Values below 0.05 are highlighted in bold.

celltype	All	Her2	LumA	LumB	TNBC
Arterial EC	4.1480	5.7588	5.6792	5.9561	0.2513
Breast basal cell	-2.3258	-5.1388	-5.7774	9.7724	0.1604
Breast cancer specific luminal cell	0.4963	0.4725	0.2547	-0.1758	**4.8301**
Breast cancer specific proliferation luminal cell	0.5299	3.1262	-2.8391	3.0240	-0.4823
Capillary EC	2.7170	-1.6411	**8.3402**	-2.0976	-3.3801
CD4 T	-0.5431	-0.4632	**-1.8841**	0.2566	1.9773
cDC2	-6.1000	-3.0669	-8.3753	-7.8065	-3.5097
CFD fibroblast	0.9607	-9.8444	0.7959	5.7762	1.2315
CXCL1/2/3 fibroblast	**14.4911**	-2770.3697	-57.4716	-45.1035	**16.0235**
CXCL13 exhausted CD8 T	**-7.9459**	-7.8050	-81.9326	-1.1202	-9.0308
GZMH CD8 T	-7.9645	-13.3483	-1744.1759	-14.3721	19.1266
GZMK CD8 T	-0.4547	-2.2583	**2.8079**	**-4.7062**	-0.5602
IgA plasma	-2.6353	-682.5341	8.8293	**38.5866**	-126.9289
IgG plasma cell	**-49.2270**	**-201.9568**	-270.3538	-17.4386	-31.3686
INF responsed T	-11.5394	-73.3893	12.3829	-15.9049	-18.1087
Luminal progenitor	-2.6672	1.2691	**-13.0901**	-7.3820	-0.8265
Lymphatic EC	3.1791	26.6210	**5.9853**	-1.8252	1.0355
Macrophage	0.4123	-9.4764	-3.4115	4.1899	4.9643
Mast cell	-0.2175	1.1490	-1.3997	-5.1487	**16.5062**
Monocyte	**-9.4069**	-12.5804	-4.9298	-9.4491	-9.3925
mregDC	-0.4682	-117.1112	**49.8565**	-16.2705	-25.1282
NK cell	-6.1467	-17.8020	-11.6630	-3.2864	1.8471
Other B cells	**-10.7167**	-6.4657	-22.9321	-3.9598	-16.5354
Other fibroblasts	4.3754	-25.0279	4.5560	**25.7106**	**-1446.6078**
pDC	-0.5184	2.6703	3.5524	-6.3674	-2.9030
Pericyte	0.9251	-143.1812	-2.5358	**32.5243**	-41.7658
Proliferation macrophage	8.4515	-5.2617	20.6023	10.6069	8.7878
Proliferation T/NK	-3.0635	-68.8427	**48.0098**	**20.3554**	-8.0451
Smooth muscle cell	0.9353	-7.3686	5.5805	5.1091	-5.5453
Tfh	-8.4608	13.6374	-14.1236	-19.0414	-15.0472
TGM2 luminal cell	**1.9472**	**6.5208**	**2.4496**	0.1378	-0.7194
TNBC-specific epithelial cell	0.7026	17.1293	NaN	NaN	0.2678
Treg	**6.4156**	4.5115	3.9496	**10.2100**	1.5692
Venous EC	-3.1182	-3.7308	-1.8189	1.4798	-10.6539

Table S55: **Coefficients of the Cox proportional hazard model** for all Music sub celltypes. Coefficients having a p-Value below 0.05 are highlighted in bold.

celltype	All	Her2	LumA	LumB	TNBC
Arterial EC	0.0536	0.1743	0.0935	0.3963	0.9486
Breast basal cell	0.3481	0.7767	0.1572	0.5831	0.9617
Breast cancer specific luminal cell	0.4334	0.7861	0.7888	0.8398	**0.0010**
Breast cancer specific proliferation luminal cell	0.5934	0.3374	0.2442	0.0779	0.7713
Capillary EC	0.1513	0.7683	**0.0011**	0.6290	0.4239
CD4 T	0.3511	0.7705	**0.0295**	0.8307	0.1237
cDC2	0.0782	0.7065	0.1129	0.4432	0.5724
CFD fibroblast	0.6723	0.2381	0.7851	0.2427	0.8242
CXCL1/2/3 fibroblast	**4.00e-5**	0.3142	0.3647	0.3786	**3.12e-13**
CXCL13 exhausted CD8 T	**0.0301**	0.3710	0.0517	0.8506	0.1047
GZMH CD8 T	0.3616	0.3908	0.2612	0.4398	0.3088
GZMK CD8 T	0.6879	0.5224	**0.0428**	**0.0115**	0.8540
IgA plasma	0.9521	0.0913	0.8950	**7.00e-5**	0.2221
IgG plasma cell	**0.0372**	**0.0319**	0.0621	0.6270	0.1707
INF responsed T	0.1583	0.1636	0.3398	0.2773	0.3050
Luminal progenitor	0.1226	0.6494	**0.0278**	0.2075	0.7029
Lymphatic EC	0.1356	0.0646	**0.0461**	0.7770	0.7703
Macrophage	0.8924	0.3540	0.5759	0.5393	0.2721
Mast cell	0.9303	0.9743	0.5860	0.5081	**0.0006**
Monocyte	**0.0308**	0.3181	0.7094	0.3937	0.0843
mregDC	0.9780	0.0650	**0.0025**	0.4981	0.5158
NK cell	0.3874	0.4007	0.4262	0.8355	0.8505
Other B cells	**0.0333**	0.7130	0.0599	0.4162	0.0998
Other fibroblasts	0.4675	0.4468	0.6278	**0.0272**	**1.86e-05**
pDC	0.8878	0.8065	0.5257	0.3719	0.7480
Pericyte	0.9314	0.1635	0.9286	**0.0122**	0.0559
Proliferation macrophage	0.1191	0.6952	0.0733	0.3766	0.3300
Proliferation T/NK	0.5410	0.0819	**0.0017**	**0.0282**	0.2274
Smooth muscle cell	0.8360	0.5979	0.5088	0.5752	0.4228
Tfh	0.2064	0.2488	0.1293	0.4386	0.4510
TGM2 luminal cell	**0.0096**	**0.0362**	**0.0061**	0.9271	0.8862
TNBC-specific epithelial cell	0.8335	0.1608	NaN	NaN	0.9384
Treg	**0.0201**	0.5308	0.4446	**0.0110**	0.7881
Venous EC	0.3797	0.6876	0.6786	0.8766	0.3185

Table S56: **p-values of the Cox proportional hazard model** for all Music sub celltypes. p-Values below 0.05 are highlighted in bold.

7 TCGA Benchmark summary

Method	Layer	All	Her2	LumA	LumB	TNBC	Total
HIDE	Major	0	4	1	2	2	9
	Minor	1	5	2	2	3	13
	Sub	4	6	4	7	6	27
BayesPrism	Major (ILA)	0	3	0	2	2	7
	Major (sum. apr.)	0	3	1	0	0	4
	Minor (ILA)	1	3	0	2	3	9
	Minor (sum. apr.)	2	2	1	2	1	8
	Sub	3	7	2	6	6	24
CIBERSORTx	Major (ILA)	1	4	1	1	0	7
	Major (sum. apr.)	2	1	2	0	0	5
	Minor (ILA)	0	3	4	2	0	9
	Minor (sum. apr.)	2	1	3	1	0	7
	Sub	4	1	11	4	2	22
Music	Major (ILA)	2	2	3	3	0	10
	Major (sum. apr.)	3	1	2	1	0	7
	Minor (ILA)	2	3	3	3	1	12
	Minor (sum. apr.)	3	1	3	2	1	10
	Sub	7	2	8	6	4	27

Table S57: **Summary of significant p-Values of the TCGA-BRCA survival analysis per layer.** "Sum. apr." refers to the summing approach, "ILA" to the individual level approach.

	BayesPrism		CIBERSORTx		MuSiC		HIDE
	sum. apr.	ILA	sum. apr.	ILA	sum. apr.	ILA	
inconsistent	0	3	0	3	0	7	0
consistent	4	4	4	5	10	7	15

Table S58: **Number of consistensies and inconsistensies between the cell type levels for the cell types significant for survival.** All cell types which are identical between major, minor and sub-minor are regarded or cell types which just have one child term. We count an inconsistency when a cell type is significant in one level but the same cell type is not significant in another level or has a different sign. Cell types are counted consistent when they are significant in two levela and have the same sign.

	BayesPrism	CIBERSORTx	Music	HIDE
major level	4	1	6	5
minor level	2	2	2	6
sub level	7	3	8	11
total	13	6	16	22

Table S59: Count of significant celltypes across the breast cancer subtypes linked in a chain. The major and minor levels of BayesPrism, CIBERSORTx and Music have been estimated by the independent level approach. A chain is defined by a significant celltype that has either a significant parent or child celltype. In the case of a celltype having only one major and various subtypes, but no minor celltype, the chain is defined as a significant in a parent cell type in at least one of its corresponding child cell types.

	BayesPrism	CIBERSORTx	Music	HIDE
major level	3	4	3	5
minor level	2	3	2	6
sub level	5	9	7	11
total	10	16	12	22

Table S60: **Count of significant celltypes of the TCGA-BRCA survival analysis across the breast cancer subtypes linked in a chain**. The major and minor levels of BayesPrism, CIBERSORTx and Music have been estimated by the summing approach. In the case of a celltype having only one major and various subtypes, but no minor celltype, the chain is defined as a significant celltype in a parent cell type and at in at least one of its corresponding child cell types.
